# Associations of self-reported and objectively measured physical activity and amyotrophic lateral sclerosis risk

**DOI:** 10.1136/jnnp-2026-339165

**Published:** 2026-08-13

**Authors:** Jiali Gao, Christos V Chalitsios, Derrick Bennett, Aiden Doherty, Martin R Turner, Alexander G Thompson

**Affiliations:** 1 Nuffield Department of Clinical Neurosciences, University of Oxford, Oxford, UK; 2 Nuffield Department of Population Health, Oxford University, Oxford, UK

**Keywords:** MOTOR NEURON DISEASE, GENETICS, NEUROGENETICS, C9ORF, EPIDEMIOLOGY

## Abstract

**Background:**

The role of physical activity in the risk of amyotrophic lateral sclerosis (ALS) is debated. It is also unclear whether the association differs in people at high genetic risk of ALS.

**Methods:**

The strength and shape of the association between self-reported and device-measured physical activity and incident diagnosis of ALS in the UK Biobank cohort was analysed using Cox regression, adjusting for potential confounders. Cubic splines were used to assess non-linearity. Analyses were performed in the entire cohort and restricted to those with increased genetic risk due to *C9ORF72* expansion carriage or C-allele homozygosity at rs12608932 in *UNC13A*.

**Results:**

Among 384 836 participants with valid questionnaire data, the median age at recruitment was 57.0 years (IQR 50.0–63.0) and median follow-up was for 14.0 years (IQR 13.3–14.6), with 541 incident diagnoses of ALS. Higher self-reported physical activity was associated with a lower risk of ALS (HR_high vs low_=0.77, 95% CI 0.61 to 0.96). The relationship was non-linear, with lowest risk in those in the mid-range self-reported activity. Higher overall device-measured activity was also associated with a lower risk of ALS (HR_per 1SD_ = 0.75, 95% CI 0.58 to 0.97, n=96 570, 98 ALS events) but with a linear dose-response relationship. The association of physical activity with ALS was similar in individuals with C-allele homozygosity at rs12608932 in *UNC13A* and directionally consistent but not statistically significant in *C9ORF72-*HRE carriers (n=535, 56 ALS events).

**Conclusion:**

Higher self-reported and device-measured overall physical activity were associated with a lower risk of ALS overall, but with a potentially non-linear dose-response relationship.

**WHAT IS ALREADY KNOWN ON THIS TOPIC**

- Multiple studies have linked strenuous exercise to higher ALS risk in highly selected populations, such as specific athlete groups, but several population-based studies failed to find a positive association between physical activity and ALS risk, and some have even found an inverse association. Moreover, although intervention to prevent ALS is most feasible in carriers of ALS-associated genetic variants, only one study investigated the effect of physical activity on ALS risk in this group, using retrospective analysis in 17 *C9ORF72* expansion carriers.

**WHAT THIS STUDY ADDS**

- This study incorporates data from a large population-based prospective cohort of 384 836 individuals to add to a growing body of evidence that higher physical activity levels in the general population are associated with a lower risk of ALS. This study incorporates both subjectively reported and objective device measurements of physical activity in investigating risk of ALS and to assess the impact of physical activity in a population-based cohort of *C9ORF72* expansion carriers.

**HOW THIS STUDY MIGHT AFFECT RESEARCH, PRACTICE OR POLICY**

- These results reiterate the wide-ranging benefits of physical activity and do not support its avoidance in individuals at higher genetic risk of ALS.

## Introduction

 Amyotrophic lateral sclerosis (ALS) is characterised by progressive neurodegeneration of the corticomotorneuronal system, overlapping clinically, genetically and pathologically with frontotemporal dementia (FTD). Genetic factors explain a large portion of ALS risk, with well-characterised contributions from large effect rare variants and small effect common variants.^1^ Of these, the most common rare variant cause of ALS and FTD in European-ancestry populations is an intronic hexanucleotide repeat expansion (HRE) in *C9ORF72*, identifiable in 10% of all ALS cases,^2^ while CC homozygosity at rs12608932 in the *UNC13A* gene (present in 13% of people of European ancestry) is associated with a doubling of ALS risk.^3^ The factors driving age of onset and susceptibility in both monogenetic and non-monogenetic cases remain poorly understood but are presumed to reflect the interaction of a susceptible genetic background with stochastic events in the context of nervous system ageing.^2^

The influence of modifiable risk factors on ALS risk remains unclear. Identifying such factors is especially salient to individuals carrying ALS-associated monogenic variants where preventative intervention is most feasible. Traditionally, male sex and advancing age^4^ were the only well-established epidemiological risk factors, but evidence also implicates lower body mass index (BMI)^5 6^ and changes in lipid profiles many years prior to symptom onset.^7 8^ The lasting association of ALS with the eponymous USA baseball player Lou Gehrig and other high-profile athletes has fuelled speculation over the role of physical activity in ALS pathogenesis. However, the evidence remains mixed,^9^ and a recent Norwegian study suggested that higher levels of physical activity may instead be protective.^10^ Many existing studies focus on strenuous exercise and all rely primarily on questionnaire measures subject to recall bias. Meanwhile, evidence for an association between physical activity and ALS in the *C9ORF72-*HRE carrier group is confined to retrospective data in 17 participants.^11^

In contrast, wearable accelerometer devices allow the objective measurement of total physical activity, including occupational, domestic and incidental activity,^12^ across large-scale population cohorts. In this study, we aimed to quantify the association between physical activity, measured by wrist-worn accelerometry, as well as subjective questionnaires, and ALS incidence, in a prospective cohort of almost 400 000 UK Biobank participants. Crucially, in order to explore interactions between genetics and physical activity on ALS risk, we performed subgroup analysis in 701 people at higher risk of ALS due to carrying the *C9ORF72*-HRE (identified using ExpansionHunter) and in those with C-allele homozygosity at rs12608932 in *UNC13A*. We also explored interactions with other ALS risk factors, such as BMI and lipid measures.

## Materials and methods

This study follows Strengthening the Reporting of Observational Studies in Epidemiology (STROBE) guidelines (online supplemental information).

### Study population

The UK Biobank is a population-based prospective cohort study of 502 625 participants, aged 40–69 at recruitment between 2006 and 2010.^13^ Approximately 9.2 million people living<40 km from an assessment centre were invited (response rate 5.5%).

### Physical activity assessment

#### Subjective

Physical activity was assessed using the short International Physical Activity Questionnaire (IPAQ) at baseline and processed according to IPAQ guidelines.^14^ Participants reported the frequency (days/week) and duration (minutes/day) of walking, moderate and vigorous-intensity physical activity in the preceding week. Metabolic equivalent of task (MET) minutes per week were calculated by summing time spent in each activity category weighted by its energy expenditure. Results were transformed by cube root to approximate a normal distribution and converted to Z-scores for easier interpretation. Total physical activity was also analysed categorically, grouping into low, moderate and high categories using IPAQ criteria (online supplemental methods).

A dichotomous variable of ‘strenuous sports and other exercise’, defined as at least 2 to 3 days per week of self-reported ‘strenuous sports’ or ‘other exercises’ for at least 15–30 min, was created for comparison with previous reports.^11^

#### Objective

An Axivity AX3 triaxial accelerometer (Axivity Ltd., Newcastle, UK) was worn by 103 687 participants on their dominant wrist for 7 days in 2013–2015. Participants with documented email addresses were randomly invited (n=236 519, response rate 44.8%). After excluding individuals whose data could not be calibrated, with >1% clipped values, implausibly high activity (overall acceleration vector>100 *mg*), or insufficient wear time (<72 hours of data or missing data for any 1 hour period of the average 24-hour cycle), 96 675 were left for analysis.^15^

Overall physical activity (‘overall acceleration’) was summarised as Z-scores of the average vector magnitude of acceleration, a metric previously validated against doubly-labelled water physical activity energy expenditure.^15 16^ Proportions of time spent in sedentary (≤1.5 METs), light (1.5–3 METs), moderate-to-vigorous (≥3 METs) physical activity and sleep were estimated from accelerometry by machine learning models trained on 152 free-living individuals, with wrist-worn accelerometry data and labels of activity captured using wearable cameras and time-use diaries.^17^

### Covariate assessment

At the baseline assessment, sociodemographic, lifestyle and health-related factors were collected by touch-screen questionnaire and computer-assisted interview. Additionally, BMI and serum lipoprotein levels were measured using the Beckman Coulter AU5800 platform. UK Biobank data fields used in this study are listed in online supplemental table 1.

### Genetic data

Whole-genome sequencing was performed on 490 640 participants using Illumina NovaSeq technology on blood samples taken at baseline. *C9ORF72* repeat size was estimated using ExpansionHunter (online supplemental methods).^18^ Genotyping was also performed using Affymetrix (now part of ThermoFisher Scientific), from which rs12608932 genotype was extracted (online supplemental methods).

### Outcome ascertainment

Incident ALS cases were extracted from linked Hospital Episode Statistics (HES) or death registry data across England, Wales and Scotland using the International Classification of Diseases ninth (ICD-9: 335.2) and tenth (ICD-10: G12.2) revision codes. Prevalent ALS cases at the time of physical activity measurement, identified from these sources and self-report, were excluded.

### Statistical analysis

Cox regression models were used to estimate the HR of developing ALS, with age as the underlying time scale. Follow-up time was defined from birth to first recorded ALS diagnosis or censoring. Individuals recorded to have died were censored at their date of death (data ends 31/08/2024 in England and Wales and 30/11/2024 in Scotland), and those alive were censored at end of hospital episode data follow-up (31/03/2023 in England, 31/8/2022 in Scotland, 31/5/2022 in Wales). This approach assumes that individuals dying with ALS will have this captured on their death certificate in order to maximise case ascertainment.

The primary analysis was adjusted for predefined covariates: sex, smoking status (never, previous, current), alcohol consumption (never, <3 times per week, ≥3 times per week), age completing full-time education (years), employment status (employed, not employed), townsend deprivation index and BMI (online supplemental table 1). The analysis in carriers of the *C9ORF72-*HRE was adjusted by age and sex only due to lower sample size and power (online supplemental table 2).

Dose-response relationships between physical activity and ALS risk were assessed using restricted cubic spline models, with the median as the reference point and knots at the 5th, 50th and 95th centiles to minimise the impact of outliers. These non-linear models were then compared with the linear model using the likelihood ratio test (LRT).

Analyses were stratified by age (<50, 50–59, 60–69) and sex, and were repeated in only individuals reporting white ethnicity to investigate for subgroup-specific effects. Other ethnicities accounted for 5.4% of the cohort and were not analysed individually.

Several sensitivity analyses were conducted. The model was sequentially adjusted by removal of BMI to assess the impact of low-level collinearity between BMI and physical activity; addition of low density lipoprotein (LDL) and high density lipoprotein (HDL), previously linked to ALS risk, to assess for potential mediation by these lipids; addition of self-reported health (poor, fair, good and excellent), medication number, cardiovascular disease (hypertension, stroke, myocardial infarction or angina) and sedentary hours (sum of self-reported time spent watching TV, using a computer and driving per day) to assess for potential confounding by these factors. To assess for reverse causality, individuals with an ALS diagnosis or censorship event within 2, 4, 6 and 8 years of questionnaire assessment and within 2, 4 and 6 years of accelerometry were sequentially excluded. To remove the influence of hospital admission frequency which may lead to ascertainment bias, analyses were repeated using only death registry data, considered likely to be of higher accuracy, and repeated excluding individuals with pre-existing cardiovascular disease or self-reporting poor health. Multiple imputation by chained equations was also used to assess the impact of missing covariate data, assuming data to be missing at random.^19^ The proportion of missing data ranged from 0–14% (online supplemental table 3). Full details are provided in online supplemental methods and online supplemental figures 8–11.

Approximate power calculations were performed for Cox regressions of high versus low IPAQ category and for overall acceleration, adjusted by age only and are found in online supplemental table 2.

Analyses were conducted using R V. 4.3.1.

## Results

### Baseline characteristics

Out of 501 936 participants, 384 836 (52.1% females) had valid physical activity questionnaire data, with a median age of 57.0 years (IQR 50.0–63.0) (table 1). Over a median follow-up of 14.0 years (IQR 13.3–14.6), there were 541 incident ALS diagnoses (449 from hospital episode statistics, 91 from death data and one from self-report). Among 96 570 participants (56.3% female) with valid accelerometry data, 98 incident ALS diagnoses were recorded during a median follow-up of 14.0 years (IQR 13.3–14.6).

**Table 1:** Baseline characteristics by data availability

	Questionnaire (N=3 84 836)	Accelerometer (N=96 570)	*C9ORF72* HRE carriers - Questionnaire (N=535)	*C9ORF72* HRE carriers - Accelerometer (N=138)	CC genotype at rs12608932 – Questionnaire (N=46 872)	CC genotype at rs12608932 – Accelerometer (N=3029)
Age at recruitment (yrs)						
Median (IQR)	57.0 (50.0, 63.0)	57.0 (50.0, 62.0)	54.0 (47.0, 62.0)	53.0 (47.0, 58.8)	57.0 (49.0, 63.0)	57.0 (50.0, 62.0)
Sex						
Male	184 349 (47.9 %)	42 196 (43.7 %)	244 (45.6 %)	63 (45.7 %)	22 429 (47.9 %)	5142 (43.4 %)
Female	200 487 (52.1 %)	54 374 (56.3 %)	291 (54.4 %)	75 (54.3 %)	24 443 (52.1 %)	6718 (56.6 %)
Ethnicity						
Non-white	18 793 (4.9 %)	2979 (3.1 %)	6 (1.1 %)	1 (0.7 %)	3193 (6.8 %)	563 (4.7 %)
White	364 935 (94.8 %)	93 256 (96.6 %)	527 (98.5 %)	137 (99.3 %)	43 530 (92.9 %)	11 256 (94.9 %)
Body Mass Index (kg/m2)						
Median (IQR)	26.6 (24.1, 29.7)	26.0 (23.6, 29.0)	25.2 (22.9, 28.6)	25.0 (23.0, 27.5)	26.5 (24.0, 29.6)	26.0 (23.6, 29.0)
Smoking status						
Never	210 746 (54.8 %)	55 011 (57.0 %)	340 (63.6 %)	79 (57.2 %)	26 174 (55.8 %)	6831 (57.6 %)
Previous	134 401 (34.9 %)	34 624 (35.9 %)	154 (28.8 %)	51 (37.0 %)	15 891 (33.9 %)	4179 (35.2 %)
Current	38 723 (10.1 %)	6677 (6.9 %)	38 (7.1 %)	8 (5.8 %)	4683 (10.0 %)	822 (6.9 %)
Alcohol consumption frequency						
Never	27 946 (7.3 %)	5473 (5.7 %)	52 (9.7 %)	7 (5.1 %)	3585 (7.6 %)	713 (6.0 %)
<3 times/week	181 661 (47.2 %)	43 868 (45.4 %)	280 (52.3 %)	71 (51.4 %)	22 068 (47.1 %)	5392 (45.5 %)
3+times/week	175 046 (45.5 %)	47 148 (48.8 %)	203 (37.9 %)	60 (43.5 %)	21 195 (45.2 %)	5745 (48.4 %)
Townsend deprivation index						
Median (IQR)	−2.23 (-3.70, 0.330)	−2.44 (-3.81, −0.170)	−2.12 (-3.72, 0.540)	−2.57 (-3.88, 0.908)	−2.21 (-3.68, 0.350)	−2.42 (-3.80, −0.0800)
Age completed full time education (yrs)						
Median (IQR)	18.0 (16.0, 21.0)	20.0 (16.0, 21.0)	18.0 (16.0, 21.0)	18.0 (16.0, 21.0)	18.0 (16.0, 21.0)	20.0 (16.0, 21.0)
Employment status						
Employed	227 838 (59.2 %)	59 516 (61.6 %)	330 (61.7 %)	98 (71.0 %)	28 057 (59.9 %)	7382 (62.2 %)
Not employed	156 020 (40.5 %)	36 880 (38.2 %)	204 (38.1 %)	40 (29.0 %)	18 701 (39.9 %)	4459 (37.6 %)
Self-rated overall health						
Excellent	68 832 (17.9 %)	20 954 (21.7 %)	107 (20.0 %)	26 (18.8 %)	8396 (17.9 %)	2567 (21.6 %)
Good	224 329 (58.3 %)	57 781 (59.8 %)	329 (61.3 %)	90 (65.2 %)	27 404 (58.5 %)	7104 (59.9 %)
Fair	75 126 (19.5 %)	15 123 (15.7 %)	85 (15.9 %)	18 (13.0 %)	9102 (19.4 %)	1837 (15.5 %)
Poor	15 465 (4.0 %)	2478 (2.6 %)	14 (2.6 %)	4 (2.9 %)	1833 (3.9 %)	322 (2.7 %)

Questionnaire – participants with valid physical activity questionnaire data; accelerometer – participants with valid wrist-worn accelerometer data.

HRE, hexanucleotide repeat expansion.

Using a cut-off of 100 repeats, 699 *C9ORF72*-HRE carriers were identified, including 535 with physical activity questionnaire data, leading to 56 ALS diagnoses, and 138 carriers with accelerometry data, leading to 14 ALS diagnoses.

Measures of overall physical activity levels from the questionnaire and accelerometry were only weakly correlated (Pearson R 0.24, p<0.001, online supplemental figure 1).

### Self-reported physical activity was associated with lower ALS risk

Using complete-case data, individuals in the middle IPAQ category had 26% lower risk of ALS compared with those in the low category (HR=0.74, 95% CI 0.59 to 0.94) and those in the high IPAQ category had a 23% lower risk (HR=0.77, 95% CI 0.61 to 0.96; p_trend_=0.062; figure 1A). Self-reported overall physical activity modelled continuously as MET minutes per week was not significantly associated with ALS risk (HR_per SD_=0.96, 95% CI 0.88 to 1.05; figure 1B), though cubic spline models suggested a non-linear association, with higher ALS risk at low activity levels (figure 2A, LRT p_non-linearity_=0.001). Associations between self-reported physical activity and ALS risk differed by strenuousness, with an association observed only for the middle quartiles of moderate physical activity (HR_Q1-Q2_=0.73, 95% CI 0.58 to 0.92; HR_Q1-Q3_=0.77, 95% CI 0.60 to 0.98; HR_Q1-Q4_=0.80, 95% CI 0.64 to 1.01), but not for walking (HR_Q1-Q2_=0.79, 95% CI 0.63 to 1.00; HR_Q1-Q3_=0.85, 95% CI 0.67 to 1.08; HR_Q1-Q4_=0.98, 95% CI 0.76 to 1.26) or vigorous activity (HR_Q1/2-Q3_=0.87, 95% CI 0.71 to 1.07; HR_Q1/2-Q4_=0.90, 95% CI 0.72 to 1.12). Self-reported ‘Strenuous sport and other exercises’ was not associated with ALS risk (HR=0.89, 95% CI 0.73 to 1.08; online supplemental table 3 and online supplemental figure 2).

**Figure 1:**
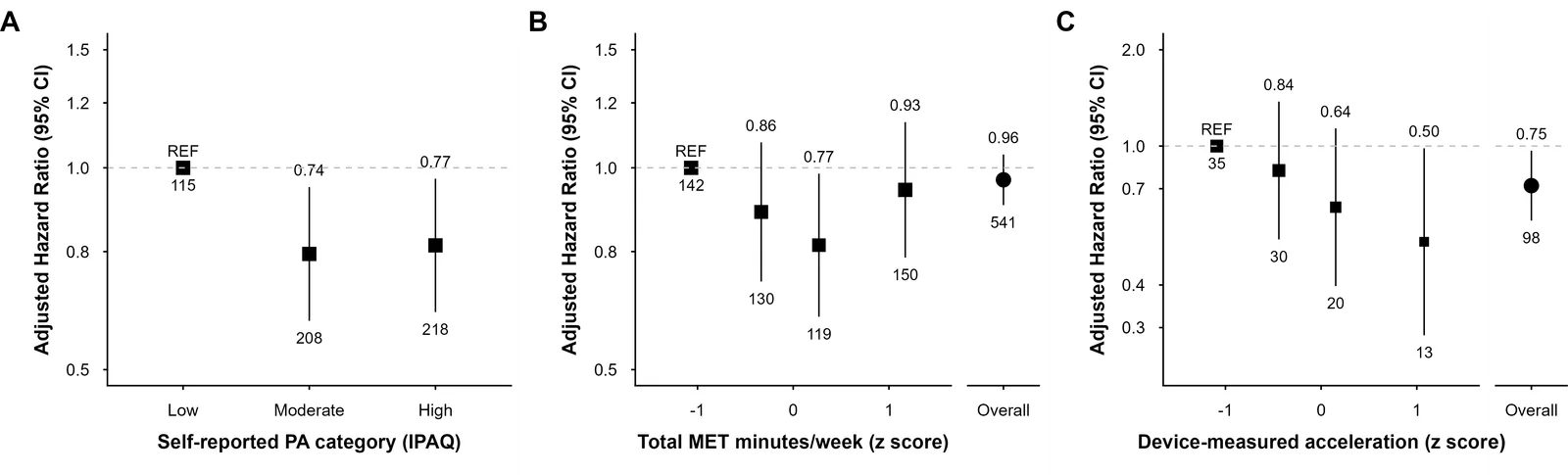
Association between overall activity measures and risk of incident ALS. HRs for ALS by (**A**) International Physical Activity Questionnaire (IPAQ) activity categories, (**B**) quartiles of metabolic equivalent task (MET) minutes/week, and (**C**) quartiles of overall acceleration, with overall HR per 1 SD increase in the activity measure when treated as a continuous variable. These were calculated using Cox regression models with age as the timescale and adjusted for sex, employment status, age leaving education, townsend deprivation index, smoking status, alcohol consumption and body mass index. The number above each vertical line is the HR and the number below each vertical line is the number of events.

**Figure 2:**
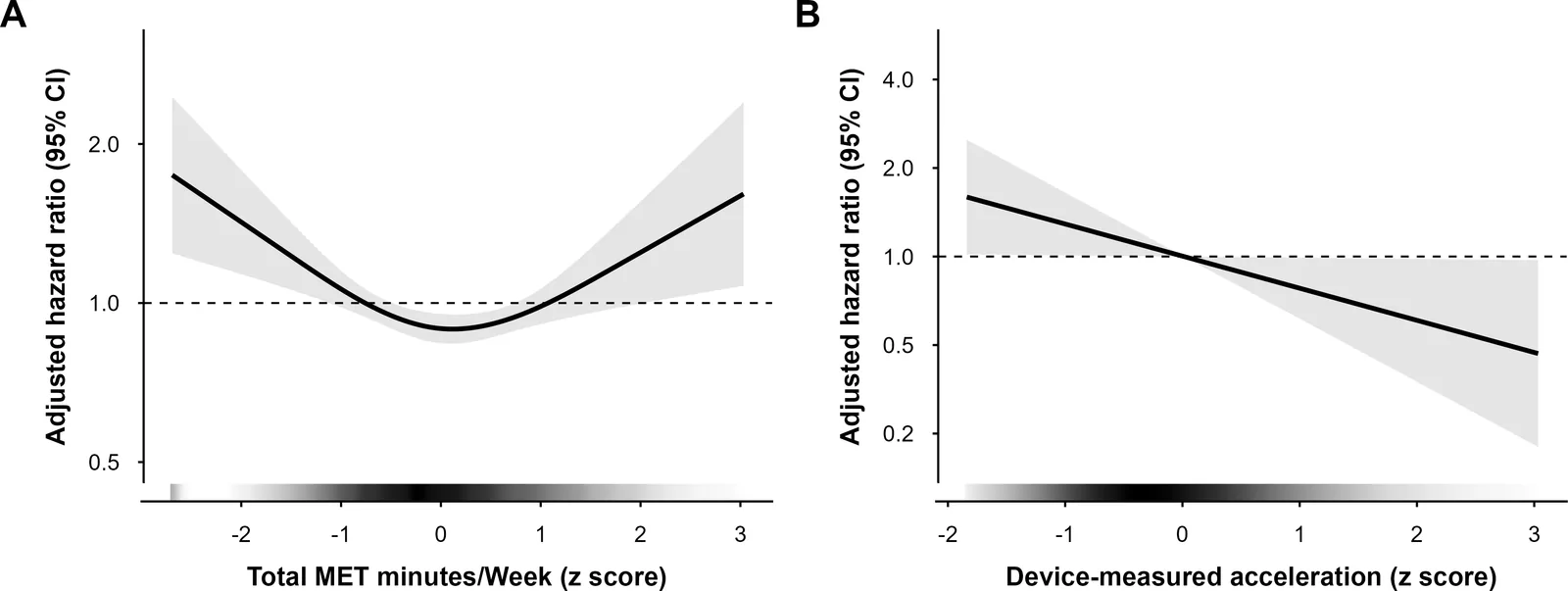
Dose-response relationships between overall activity measures and risk of incident ALS. (**A**) Dose-response relationship between MET minutes/week and risk of ALS modelled using restricted cubic splines, with knots at the 5th, 50th and 95th centiles and the reference as the median. (**B**) Dose-response relationship between overall acceleration and risk of ALS modelled using a linear Cox model (favoured over a restricted cubic spline model by likelihood ratio testing). The 95% CI band is represented in grey and the X axis rug represents the distribution of participant activity scores (black=more frequent).

### Objectively measured physical activity was associated with lower ALS risk

One SD (8.24 mg) higher overall accelerometer-measured physical activity was associated with 25% lower risk of ALS (HR=0.75, 95% CI 0.58 to 0.97, figure 1C). The dose-response association was linear (figure 2B, p_non-linearity_=0.704). Using machine learning-inferred behaviours to categorise the proportion of time spent in sedentary, light, moderate-vigorous activity and sleep, no clear relationship between activity type and ALS risk was identified, but these analyses are limited in sample size per group (online supplemental table 3 and online supplemental figure 3).

### Physical activity and ALS risk in people at higher genetic risk of ALS

Analyses in individuals carrying the *C9ORF72* expansion were limited in power (n_cases_=56 for self-reported activity and n_cases_=14 for accelerometer-derived activity, online supplemental table 2). Neither self-reported physical activity nor accelerometer-measured activity were significantly associated with ALS risk. Both were directionally consistent with analyses in the whole cohort (self-reported: HR_high vs low_=0.65, 95%CI=0.33–1.31; accelerometry: HR_per SD_=0.63, 95%CI=0.36–1.11; figure 3A–C).

**Figure 3:**
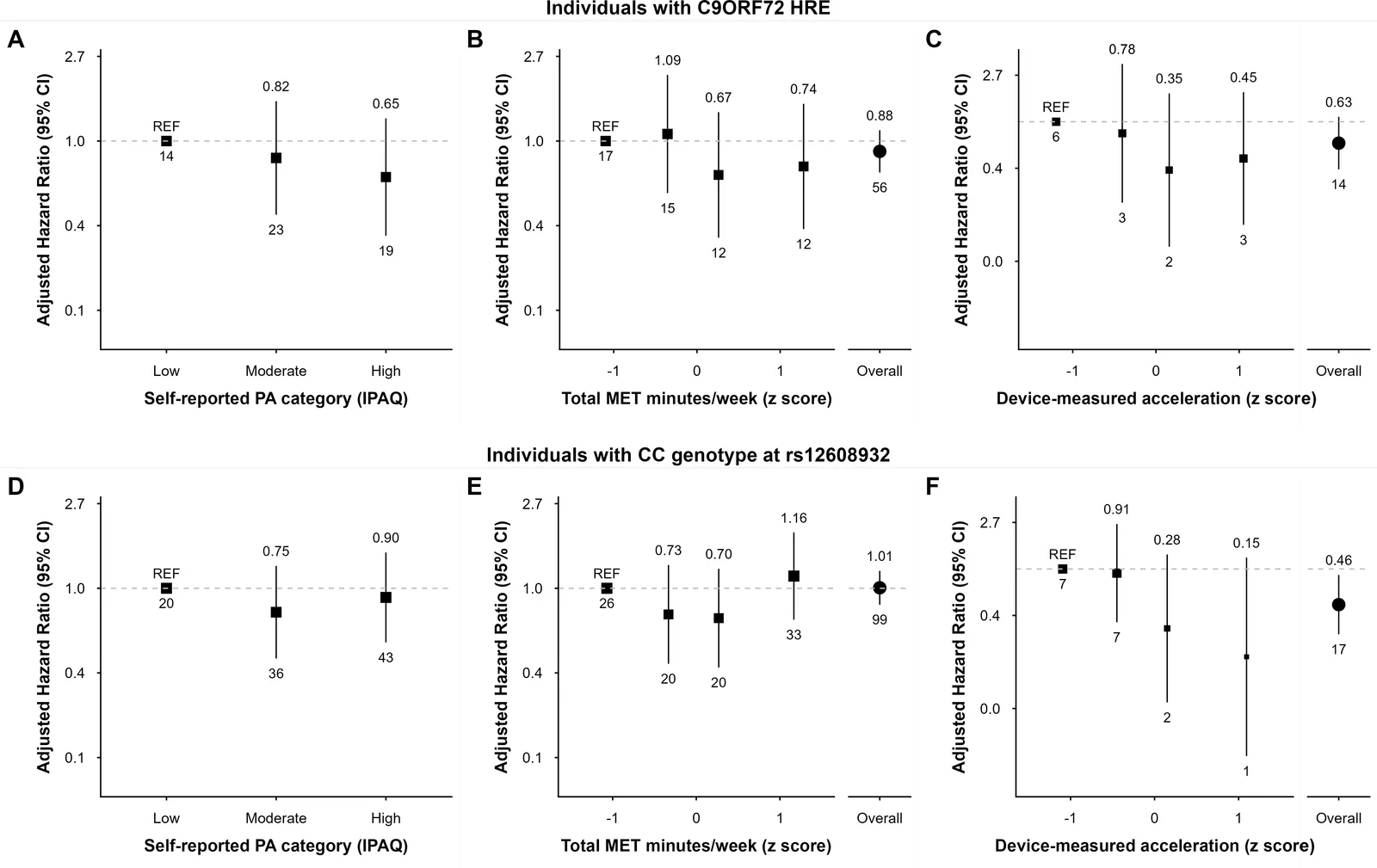
Association between physical activity measures and risk of incident ALS in those at increased genetic risk. HRs for ALS in *C9ORF72* hexanucleotide repeat expansion carriers (**A–C**) and those with C-allele homozygosity at UNC13A rs12608932 (**D–F**) by International Physical Activity Questionnaire (IPAQ) activity categories (**A, D**), quartiles of metabolic equivalent task (MET) minutes/week (**B, E**), and quartiles of overall acceleration, with overall HR per 1 SD increase in the activity measure when treated as a continuous variable (**C, F**). These were calculated using Cox models with age as the timescale and adjusted for sex, employment status, age leaving education, townsend deprivation index, smoking status, alcohol consumption and body mass index. The number above each vertical line is the HR and the number below each vertical line is the number of events.

Meanwhile, in individuals carrying the common CC risk variant at rs12608932 (n_cases_=99 for self-reported activity and n_cases_=17 for accelerometer-derived activity), one SD (8.24 mg) higher overall accelerometer-measured activity was associated with a HR of 0.46 (95%CI 0.25 to 0.88) of ALS, whereas self-reported physical activity was not significantly associated with ALS risk (figure 3D–F).

### Subgroup analyses

The observed associations of ALS risk with IPAQ category and overall acceleration in the whole cohort were similar across age categories, but the favourable association was more prominent in female than male participants (p for interaction=0.054 for IPAQ category and 0.039 for overall acceleration). The results of analyses restricted to individuals reporting white ethnicity remained largely unchanged (online supplemental figure 4).

### Sensitivity analyses

Models constructed separately excluding BMI, including HDL and LDL cholesterol and including measures of comorbidity (overall health, cardiovascular disease, number of medications and sedentary activity) yielded effect estimates consistent with the primary analysis. The inclusion of lipid measures in the models yielded effect estimates that were similar in direction and magnitude to the primary analysis, but no longer statistically significant, likely owing to the reduced sample size resulting from missing lipid data. (online supplemental table 4). All analyses were repeated with multiple imputation of missing values, yielding parameter estimates consistent with the complete case primary analysis (online supplemental table 5).

To assess for reverse causality, individuals with an ALS diagnosis or censorship event within 2, 4, 6, and 8 years of questionnaire measurement and within 2, 4, and 6 years of accelerometry were sequentially excluded. The magnitude of the association remained similar, and even at 8 years, the association with IPAQ activity category remained significant (HR=0.70, 95% CI 0.52 to 0.94, online supplemental figure 5), although at 6 and 8 years, sample size and power were limited, especially for accelerometry analyses. To remove the influence of hospital admission frequency on ALS diagnoses (due to collider bias), the analyses were repeated using only death registry data. Despite this lower-powered analysis (online supplemental table 2), both self-reported and overall accelerometer-measured physical activity was associated with fewer ALS deaths (self-reported: HR_high vs low_=0.77, 95%CI=0.59–0.99, 460 events; accelerometry: HR_per SD_=0.72, 95% CI 0.55 to 0.96, 83 events) (online supplemental figure 6). Finally, exclusion of individuals with premorbid cardiovascular disease and individuals self-reporting poor health at baseline did not materially alter the results (online supplemental figure 7).

## Discussion

Using both self-reported and device measured physical activity in a large prospective cohort, this study demonstrated a robust association between higher physical activity and lower ALS risk, which is more prominent in women and related to levels of moderate activity as opposed to vigorous activity or walking. The favourable association of device-measured activity was also seen in individuals with a common risk variant in *UNC13A* and was directionally consistent though not statistically significant in a population-based cohort of *C9ORF72* repeat expansion carriers.

Using a large-scale prospective study design with longitudinal follow-up, our findings converge with those of a recent large-scale Norwegian cohort study linking higher reported physical activity with lower ALS risk,^10^ although in the Norwegian study this association was observed exclusively in male participants. Our study suggests the association is more prominent in females; this discrepancy may relate to the differing ages (and hence menopausal statuses) of the two cohorts, with the UK Biobank having a generally older age at recruitment (average age of 57.0 years compared with 40.7 years in the Norwegian study). Female reproductive hormones are associated with lower ALS risk^20^ and are affected by physical activity,^21^ which may explain this link. Indeed, in the *SOD1* mouse model of ALS, exercise was shown to delay disease onset and improve survival in females, with associated oestrus cycle changes.^22^

Other previous reports have often linked higher physical activity levels with higher risk of ALS. These studies have typically focused on highly selected populations with extreme levels of strenuous activity levels or have used retrospective data prone to recall bias. Higher ALS incidence has been reported among varsity athletes and soccer players,^23^ but these findings are potentially confounded by head trauma, which is itself inconsistently associated with ALS.^24 25^ Moreover, while case-control studies have linked historical physical activity with higher ALS risk^26^ and earlier ALS onset,^27^ cohort studies have reported more mixed results.^28 29^ Mendelian randomisation evidence has also causally linked strenuous activity with higher ALS risk, especially in males.^11 30^ Although these findings may appear at odds with the results presented here, data are sparse at very high levels of strenuous activity, and IPAQ categories do not separate out the highest levels of activity, making firm conclusions difficult. The apparent u-shaped relationship between self-reported physical activity and ALS risk described here could be due to reverse causation or compatible with a model whereby both low and very high levels of physical activity (>9000 MET minutes per week) are associated with increased ALS risk. Such a relationship finds support in the observational literature, in which a study of competitive cross-country skiing indicated higher risk in the fastest skiers, but the slowest skiers had a lower risk than that of the general population.^31^ A similar differential relationship for levels of activity has also been identified using genetic epidemiological analysis, with light physical activity genetically correlated with lower ALS risk and moderate activity genetically correlated with higher ALS risk.^32^ Our work suggests that the risk associated with lower levels of physical activity is more relevant at the population level, and it is notable that the apparent u-shaped relationship was not observed in objectively measured activity.

The differing dose-response relationships for subjective and objective measures of physical activity with ALS risk have several potential explanations. Questionnaire data are subject to reporting bias and is weighted towards leisure-time activity, which has specifically been linked to increased ALS risk, but without a specific mechanistic explanation.^11^ Questionnaire data may also be influenced by perceived fitness and athleticism which, rather than activity, could explain the observed link between strenuous exercise and ALS.^31 33^ Conversely, accelerometry measures are better correlated with body composition,^34^ likely reflecting better capture of overall activity levels, but were recorded in a more selected population.

The contentious role of physical activity in ALS risk is particularly relevant to people carrying ALS-causing genetic variants, who must make life-altering lifestyle choices based on the limited data available. Previous evidence linking physical activity to higher ALS risk in this population was confined to retrospective data in seventeen *C9ORF72*-HRE carriers. This study, in contrast, although still limited in power, does not support forgoing the wider benefits of physical activity to mitigate ALS risk in those at higher genetic risk of ALS.

The strengths of this study include the large prospective cohort design, routine linkage to national records, the combination of device and self-reported measures of physical activity, and the integration of genetic data, allowing investigation of the interaction between physical activity and *C9ORF72* expansion status or *UNC13A* genotype on ALS risk. However, there are important limitations. Exercise measures in this study were taken at a single timepoint and do not necessarily capture lifelong exposure, although repeat accelerometry measures over time in other studies do show moderate stability.^35 36^ While we have adjusted for a wide range of possible confounders and performed sensitivity analysis excluding those with shorter follow-up, we cannot rule out potential residual confounding and reverse causation bias. Hospital and death certificate ALS diagnoses likely post-date symptom onset and may also reflect disease progression. Nonetheless, to assess the impact of possible inaccuracies and collider bias, sensitivity analyses using only death certificate diagnoses were conducted. Moreover, the phenotype and severity of ALS cases cannot be established. Finally, UK Biobank participants are known to be healthier than the general population, 94.6% report white ethnicity and all are middle-aged, limiting the generalisability of our findings in non-white ethnicities and younger-onset ALS cases.

In conclusion, higher overall physical activity levels are associated with a lower risk of ALS in the UK Biobank cohort. Overall, this result does not support the general avoidance of or reduction in physical activity, especially at levels relevant to the general population, in high ALS risk populations.

## Supplementary material

10.1136/jnnp-2026-339165 online supplemental file 1

## Data Availability

Data may be obtained from a third party and are not publicly available.
