# Supplementary material for: Associations of self-reported and objectively measured physical activity and amyotrophic lateral sclerosis risk
Source: J Neurol Neurosurg Psychiatry. 2026 Aug 13;97(11):e339165. doi: 10.1136/jnnp-2026-339165 (PMC13691708; doi:10.1136/jnnp-2026-339165)
Supplement: Supplementary file 1 [file jnnp-97-11-s001.pdf]

Higher premorbid physical activity is associated with lower risk of Amyotrophic Lateral Sclerosis Supplementary information

Jiali Gao[1], Christos V. Chalitsios[1], Derrick Bennett[2], Aiden Doherty[2], Martin R Turner[1], Alexander G Thompson[1+]

[1] Nuffield Department of Clinical Neurosciences, University of Oxford, Oxford, UK

[2] Nuffield Department of Population Health Sciences, University of Oxford, Oxford UK

CONTENTS

Supplementary methods 3
International Physical Activity Questionnaire (IPAQ) activity categories 3
Expansion Hunter 3
Multiple imputation by chained equations 3
Supplementary Table 1 – UK Biobank Data fields used in this study 5
Supplementary Table 2 – Power calculations 6
Supplementary Table 3 – Proportion of missing data for imputation 6
Supplementary Table 4 – Cox models for different physical activity variables 7
Supplementary Table 5 – Sequentially adjusted Cox models using complete case data 9
Supplementary Table 6 – Cox proportional hazard models using 100 imputed datasets 9
Supplementary Figure 1 - Association between questionnaire and accelerometer-derived overall activity measures. 10
Supplementary Figure 2 - Association between questionnaire-derived activity measures and risk of incident ALS. 11
Supplementary Figure 3 - Association between accelerometer-derived activity measures and risk of incident ALS. 12
Supplementary Figure 4 - Analysis by age category, sex and ethnicity. 13
Supplementary Figure 5 - Effect of sequential exclusion of recently diagnosed ALS patients 13
Supplementary Figure 6 - Analyses using only death certificate ALS diagnoses 14
Supplementary Figure 7 - Effect of exclusion of participants with pre-existing cardiovascular disease or self-reported poor health 14
Supplementary Figure 8 – Density plot of continuous variables for the first 5 MICE imputations in individuals with complete exercise questionnaire data 15
Supplementary Figure 9 – Density plot of continuous variables for the first 5 MICE imputations in individuals with complete accelerometery data 16
Supplementary Figure 10 – Proportion plot of categorical variables for the first 5 MICE imputations in individuals with complete exercise questionnaire data 17
Supplementary Figure 11 – Proportion plot of categorical variables for the first 5 MICE imputations in individuals with complete accelerometery data 17
References 18
STROBE Statement—checklist of items that should be included in reports of observational studies 19

Supplementary methods

International Physical Activity Questionnaire (IPAQ) activity categories

The moderate IPAQ activity category aims to capture individuals meeting the US public health recommendations of physical activity of 30 minutes or more of moderate-intensity physical activity most days.[1] However, given that the IPAQ questionnaire aims to assess physical activity across multiple domains, not just leisure time activity, the definition is adjusted accordingly. The high activity category aims to capture higher levels of participation and the low activity category consists of individuals not meeting the criteria for either of the above categories.

The exact criteria for each category are reproduced below.[2]

Low – No activity is reported OR some activity is reported but not enough to meet Categories 2 or 3.

Moderate – Either of the following three criteria:

- Three or more days of vigorous-intensity activity of at least 20 minutes per day OR
- Five or more days of moderate-intensity activity and/or walking of at least 30 minutes per day OR
- Five or more days of any combination of walking, moderate-intensity or vigorous intensity activities achieving a minimum of at least 600 MET-min/week

High – Any one of the following two criteria:

- Vigorous-intensity activity on at least three days and accumulating at least 1500 MET-minutes/week OR
- Seven or more days of any combination of walking, moderate- or vigorous-intensity activities accumulating at least 3000 MET-minutes/week

Expansion Hunter

C9ORF72 repeat expansion status was estimated using ExpansionHunter, a previously validated method of C9ORF72 repeat detection with a sensitivity of 100% and specificity of 99.8-99.9% when compared to repeat-primed PCR.[3] ExpansionHunter was run as part of the Illumina DRAGEN (Dynamic Read Analysis for GENomics) 3.7.8 pipeline using the default variant catalogue.[4] This includes 29 "off-target regions" for the *C9ORF72* repeat expansion, which are loci identified by Dolzhenko et al. where paired intra-repeat reads may misalign. Inclusion of off-target regions allows more accurate estimation of large expansion sizes beyond the fragment length, which is common with C9ORF72 expansions, but may also increase the chance of overestimating some genotypes.[3,5] Individuals in whom the lower bound of the 95% confidence interval of *C9ORF72* repeat size was over 100 repeats were classed as *C9ORF72* expansion carriers. This conservative threshold was chosen to prioritise the specificity of repeat detection.

In a random sample of 20% of the detected expansion carriers, we also estimated repeat size using ExpansionHunter v5 without inclusion of off-target regions and generated read visualisations of these results using REViewer.[6] As expected, this resulted in smaller estimations of repeat length, but all identified *C9ORF72* expansion carriers had an estimated repeat size of over 30 repeats by this method and visual inspection of REViewer read alignments suggested that all were consistent with a repeat expansion in the *C9ORF72* locus.

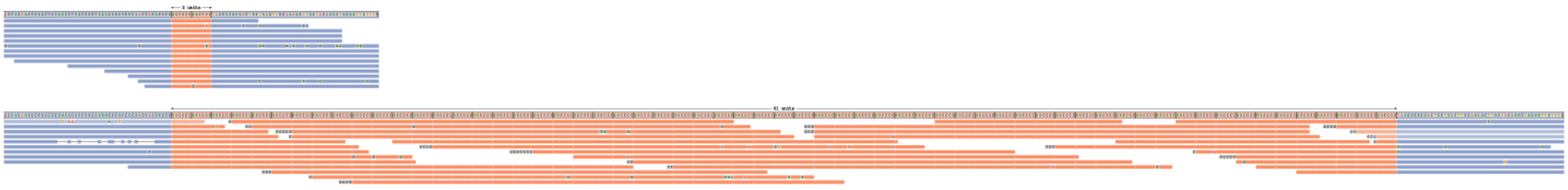

Example read visualisation

Genotyping

Genotyping was performed on 488,000 participants using two closely related purpose-designed arrays. ~50,000 participants were run on the UK BiLEVE Axiom array and the remaining ~450,000 were run on the UK Biobank Axiom array. The genotype of the single nucleotide polymorphism (SNP) rs12608932 in UNC13A was extracted from this data. The closely related SNP rs1297319 in UNC13A, which has also been linked to ALS/FTD,[7] was not included in these arrays. Given the high degree of linkage disequilibrium between these SNPs ($R^2 = 0.9536$ using LDlink[8]), imputation of this SNP was considered unlikely to add meaningful information. For quality control, individuals with mismatch between genetically determined and self-reported sex and individuals with sex chromosome aneuploidy were excluded.

Multiple imputation by chained equations

One hundred imputed datasets with ten iterations were created with the 'mice' package in R on a high-performance cluster computing system, using logistic regression for two-level factors, passive imputation from height and weight for BMI and predictive mean matching for all other variables.

The proportion of missing data for each variable is listed in **Supplementary Table 3**.

Physical activity variables were not imputed due to the high percentage of missing data (13% for questionnaire data and 81% for accelerometer data). Variables included in the imputation model (both as predictors and imputed) included Nelson-Aalen estimators, time to event, ALS diagnosis, sex, age at physical activity measurement, smoking status, alcohol consumption, age completing full-time education, employment status, Townsend Deprivation Index, BMI, height, weight, assessment centre, overall health, number of medications, presence of cardiovascular disease and sedentary hours. The variables with the highest proportion of missing data were LDL and HDL (missing in 6% and 14% of participants with activity data respectively) and reasons given for this include patient refusal, equipment malfunction, poor venous access, patient feeling unwell and technical issues with the assay. The authors identified no reasons for violation of the missing at random assumption.

Discarding ten iterations was adequate to achieve convergence and results were pooled using Rubin's rules.[9] Diagnostic plots showed good model performance (see **Supplementary Figures 8-11**)

Supplementary Table 1 – UK Biobank Data fields used in this study

**Variable**	**UKB Field ID***	**Category**	**Notes**
IPAQ activity group	22032	Exposure	
Summed MET minutes per week for all activity	22040	Exposure	
MET minutes per week for walking	22037	Exposure	
MET minutes per week for moderate activity	22038	Exposure	
MET minutes per week for vigorous activity	22039	Exposure	
Overall acceleration average	90012	Exposure	
Sleep - Overall average	40046	Exposure	
Sedentary - Overall average	40047	Exposure	
Light - Overall average	40048	Exposure	
Moderate-Vigorous - Overall average	40049	Exposure	
Sex	31	Covariate	
Year of birth	34	Covariate	
Month of birth	52	Covariate	
Age at recruitment	21022	Covariate	
End time of wear	90011	Covariate	End time of accelerometer wear – used to calculate age at accelerometery
Townsend deprivation index at recruitment	22189	Covariate	
Age completed full time education	845	Covariate	
Current employment status	6142	Covariate	Used if field 20119 is not specified
Current employment status - corrected	20119	Covariate	
Smoking status	20116	Covariate	
Alcohol intake frequency	1558	Covariate	
Body mass index (BMI)	21001	Covariate	
HDL cholesterol	30760	Covariate	
LDL direct	30780	Covariate	
Overall health rating	2178	Covariate	
Vascular/heart problems diagnosed by doctor	6150	Covariate	
Number of treatments/medications taken	137	Covariate	
Weight	21002	Covariate for imputation	
Standing height	50	Covariate for imputation	
UK Biobank assessment centre	54	Covariate for imputation	
Ethnic background	21000	Stratification variable	
Date of motor neurone disease report	42028	Outcome	
Source of motor neurone disease report	42029	Outcome	
Date lost to follow-up	191	Censoring	
Date of death	NA	Censoring	

*Further details on UKB data fields can be found at https://biobank.ndph.ox.ac.uk/showcase/index.cgi

Supplementary Table 2 – Power calculations

Variable	Cohort	N	N Cases	HRs detectable with ≥ 80% power
IPAQ category – High	Whole cohort - Model 1	375211	472	≤0.77, ≥1.30
IPAQ category – High	*C9ORF72*-HRE carriers	536	52	≤0.44, ≥2.25
IPAQ category – High	CC genotype at rs12608932	46872	99	≤0.55, ≥1.80
IPAQ category – High	Death certificate data only	375211	367	≤0.75, ≥1.33
Overall acceleration (per SD)	Whole cohort - Model 1	94495	82	≤0.74, ≥1.35
Overall acceleration (per SD)	*C9ORF72*-HRE carriers	138	14	≤0.48, ≥2.07
Overall acceleration (per SD)	CC genotype at rs12608932	11860	17	≤0.50, ≥2.0
Overall acceleration (per SD)	Death certificate data only	94495	57	≤0.72, ≥1.39

Supplementary Table 3 – Proportion of missing data for imputation

Variable	% Missing data in participants with complete exercise questionnaire data	% missing data in participants with complete accelerometery data
Sex	0	0
Age at physical activity measurement	0	0
Assessment centre	0	0
Alcohol consumption	0.05	0.08
Number of medications	0.08	0.03
TDI	0.13	0.11
Cardiovascular disease	0.14	0.15
Smoking status	0.25	0.27
Employment status	0.25	0.18
Overall health	0.28	0.24
Ethnicity	0.29	0.35
Height	0.37	0.16
Weight	0.42	0.20
BMI	0.47	0.23
Sedentary hours	1.09	1.80
Age leaving education	1.57	1.54
LDL	5.87	6.01
HDL	13.72	13.82

Supplementary Table 4 – Cox models for different physical activity variables

Variable	Category	HR (95% CI)	N	N cases
IPAQ Category	Low	REF	69209	115
IPAQ Category	Moderate	**0.74 (0.59 - 0.94)**	152910	208
IPAQ Category	High	**0.77 (0.61 - 0.96)**	152754	218
p value for trend		*0.062*		
Total MET minutes/week	Q1 (≤ 812 mins)	REF	93438	142
Total MET minutes/week	Q2 (813 – 1785 mins)	0.86 (0.68 - 1.09)	94246	130
Total MET minutes/week	Q3 (1786 – 3564 mins)	**0.77 (0.60 - 0.98)**	93811	119
Total MET minutes/week	Q4 (≥3564 mins)	0.93 (0.73 - 1.17)	93378	150
p value for trend		*0.415*		
Vigorous MET minutes/week	Q1-2* (≤ 239 mins)	REF	200614	312
Vigorous MET minutes/week	Q3 (240 – 959 mins)	0.87 (0.71 - 1.07)	95705	124
Vigorous MET minutes/week	Q4 (≥ 960 mins)	0.90 (0.72 - 1.12)	78554	105
p value for trend		*0.236*		
Moderate MET minutes/week	Q1 (≤ 119 mins)	REF	97129	153
Moderate MET minutes/week	Q2 (120 – 479 mins)	**0.73 (0.58 - 0.92)**	105666	130
Moderate MET minutes/week	Q3 (150 – 1199 mins)	**0.77 (0.60 - 0.98)**	83715	116
Moderate MET minutes/week	Q4 (≥ 1200 mins)	0.80 (0.64 - 1.01)	88363	142
p value for trend		*0.114*		
Walking MET minutes/week	Q1 (≤ 296 mins)	REF	94838	143
Walking MET minutes/week	Q2 (297 – 692 mins)	0.79 (0.63 - 1.00)	122637	161
Walking MET minutes/week	Q3 (692 – 1385 mins)	0.85 (0.67 - 1.08)	86791	124
Walking MET minutes/week	Q4 (≥ 1386 mins)	0.98 (0.76 - 1.26)	70607	113
p value for trend		*0.969*		
Strenuous sports and other exercises	No	REF	264151	400
Strenuous sports and other exercises	Yes	0.89 (0.73 - 1.08)	110722	141
Overall acceleration	Q1 (< 21.84 m*g*)	REF	20744	35
Overall acceleration	Q2 (21.84 – 26.73 m*g*)	0.84 (0.51 - 1.38)	24755	30
Overall acceleration	Q3 (26.74 – 32.33 m*g*)	0.64 (0.37 - 1.14)	24733	20
Overall acceleration	Q4 (≥ 32.34 m*g*)	**0.50 (0.26 - 0.98)**	24186	13
p value for trend		***0.027***		
Moderate-Vigorous proportion	Q1 (< 0.98%)	REF	20705	26
Moderate-Vigorous proportion	Q2 (0.98 – 2.19%)	0.80 (0.45 - 1.40)	24645	24
Moderate-Vigorous proportion	Q3 (2.20 – 3.90%)	0.79 (0.44 - 1.42)	24702	23
Moderate-Vigorous proportion	Q4 (≥ 3.91%)	0.87 (0.48 - 1.58)	24366	25
p value for trend		*0.689*		
Light proportion	Q1 (< 15.6%)	REF	20751	28
Light proportion	Q2 (15.6 – 20.1%)	1.00 (0.60 - 1.67)	24821	32
Light proportion	Q3 (20.2 – 25.1%)	0.63 (0.35 - 1.14)	24771	20
Light proportion	Q4 (≥ 25.2%)	0.63 (0.34 - 1.17)	24075	18
p value for trend		*0.059*		
Sedentary proportion	Q1 (< 33.5%)	REF	21083	18
Sedentary proportion	Q2 (33.5 – 38.8%)	0.83 (0.43 - 1.59)	24736	19
Sedentary proportion	Q3 (38.9 – 44.1%)	1.27 (0.70 - 2.30)	24781	29
Sedentary proportion	Q4 (≥ 44.2%)	1.44 (0.79 - 2.62)	23818	32
p value for trend		*0.096*		
Sleep proportion	Q1 (< 33.5%)	REF	23534	25
Sleep proportion	Q2 (33.5 – 36.4%)	0.76 (0.42 - 1.37)	25016	20
Sleep proportion	Q3 (36.5 – 40.1%)	1.01 (0.59 - 1.75)	24966	27
Sleep proportion	Q4 (≥ 40.2%)	1.07 (0.62 - 1.87)	20902	26
p value for trend		*0.591*		
Overall acceleration	Overall	**0.75 (0.58 - 0.97)**	94418	98

Adjusted for sex, employment status, age leaving education, Townsend Deprivation index, smoking status, alcohol consumption and BMI.

HR (95% CI) – Hazard ratio of ALS (95% confidence interval); N – number of individuals in this category; N cases – number of ALS cases; Q1-4 – 1st-4th quartiles; p value for trend – p value of the categorical variable when treated as an ordinal variable in cox regression. **Significant results in bold**

*The 1st and 2nd quartiles of vigorous MET minutes/week are combined because the cut-off between the two is 0

Supplementary Table 5 – Sequentially adjusted Cox models using complete case data

		Model 1			Model 2		
**Variable**	**Category**	**HR (95% CI)**	**N**	**N cases**	**HR (95% CI)**	**N**	**N cases**
IPAQ Category	Low	REF	69209	115	REF	69857	118
IPAQ Category	Moderate	**0.74 (0.59 - 0.94)**	152910	208	**0.76 (0.60 - 0.95)**	153453	209
IPAQ Category	High	**0.77 (0.61 - 0.96)**	152754	218	**0.79 (0.63 - 0.99)**	153271	220
Overall acceleration	Overall	**0.75 (0.58 - 0.97)**	94418	98	**0.78 (0.61 - 0.99)**	94610	98

		Model 3			Model 4		
**Variable**	**Category**	**HR (95% CI)**	**N**	**N cases**	**HR (95% CI)**	**N**	**N cases**
IPAQ Category	Low	REF	59694	96	REF	68146	112
IPAQ Category	Moderate	0.78 (0.61 - 1.00)	131687	181	**0.78 (0.61 - 0.98)**	151104	204
IPAQ Category	High	0.81 (0.63 - 1.04)	131998	189	0.81 (0.64 - 1.03)	150767	214
Overall acceleration	Overall	0.80 (0.61 - 1.04)	81731	84	**0.75 (0.58 - 0.97)**	93116	97

HR (95% CI) – Hazard ratio of ALS (95% confidence interval); N – number of individuals in this category; N cases – number of ALS cases. **Significant results in bold**

Model 1: adjusted for sex, employment status, age leaving education, Townsend Deprivation index, smoking status, alcohol consumption and BMI

Model 2: adjusted for sex, employment status, age leaving education, Townsend Deprivation index, smoking status, alcohol consumption (no BMI)

Model 3: adjusted for sex, employment status, age leaving education, Townsend Deprivation index, smoking status, alcohol consumption, BMI, LDL and HDL

Model 4: adjusted for sex, employment status, age leaving education, Townsend Deprivation index, smoking status, alcohol consumption, BMI, overall health, number of medications, cardiovascular disease and sedentary hours.

Supplementary Table 6 – Cox proportional hazard models using 100 imputed datasets

		Model 1	Model 2	Model 3	Model 4		
**Variable**	**Category**	**HR (95% CI)**	**HR (95% CI)**	**HR (95% CI)**	**HR (95% CI)**	**N**	**N cases**
IPAQ Category	Low	REF	REF	REF	REF	71466	126
IPAQ Category	Moderate	**0.71 (0.56-0.88)**	**0.72 (0.58-0.90)**	**0.71 (0.57-0.88)**	**0.72 (0.58-0.91)**	156360	214
IPAQ Category	High	**0.73 (0.59-0.92)**	**0.76 (0.61-0.95)**	**0.74 (0.59-0.92)**	**0.77 (0.61-0.96)**	156948	227
Overall acceleration	Overall	**0.76 (0.59-0.97)**	**0.78 (0.61-1.00)**	**0.76 (0.59-0.97)**	**0.76 (0.59-0.98)**	96548	99

HR (95% CI) – Hazard ratio of ALS (95% confidence interval); N – number of individuals in this category; N cases – number of ALS cases. **Significant results in bold**

Model 1: adjusted for sex, employment status, age leaving education, Townsend Deprivation index, smoking status, alcohol consumption and BMI

Model 2: adjusted for sex, employment status, age leaving education, Townsend Deprivation index, smoking status, alcohol consumption (no BMI)

Model 3: adjusted for sex, employment status, age leaving education, Townsend Deprivation index, smoking status, alcohol consumption, BMI, LDL and HDL

Model 4: adjusted for sex, employment status, age leaving education, Townsend Deprivation index, smoking status, alcohol consumption, BMI, overall health, number of medications, cardiovascular disease and sedentary hours.

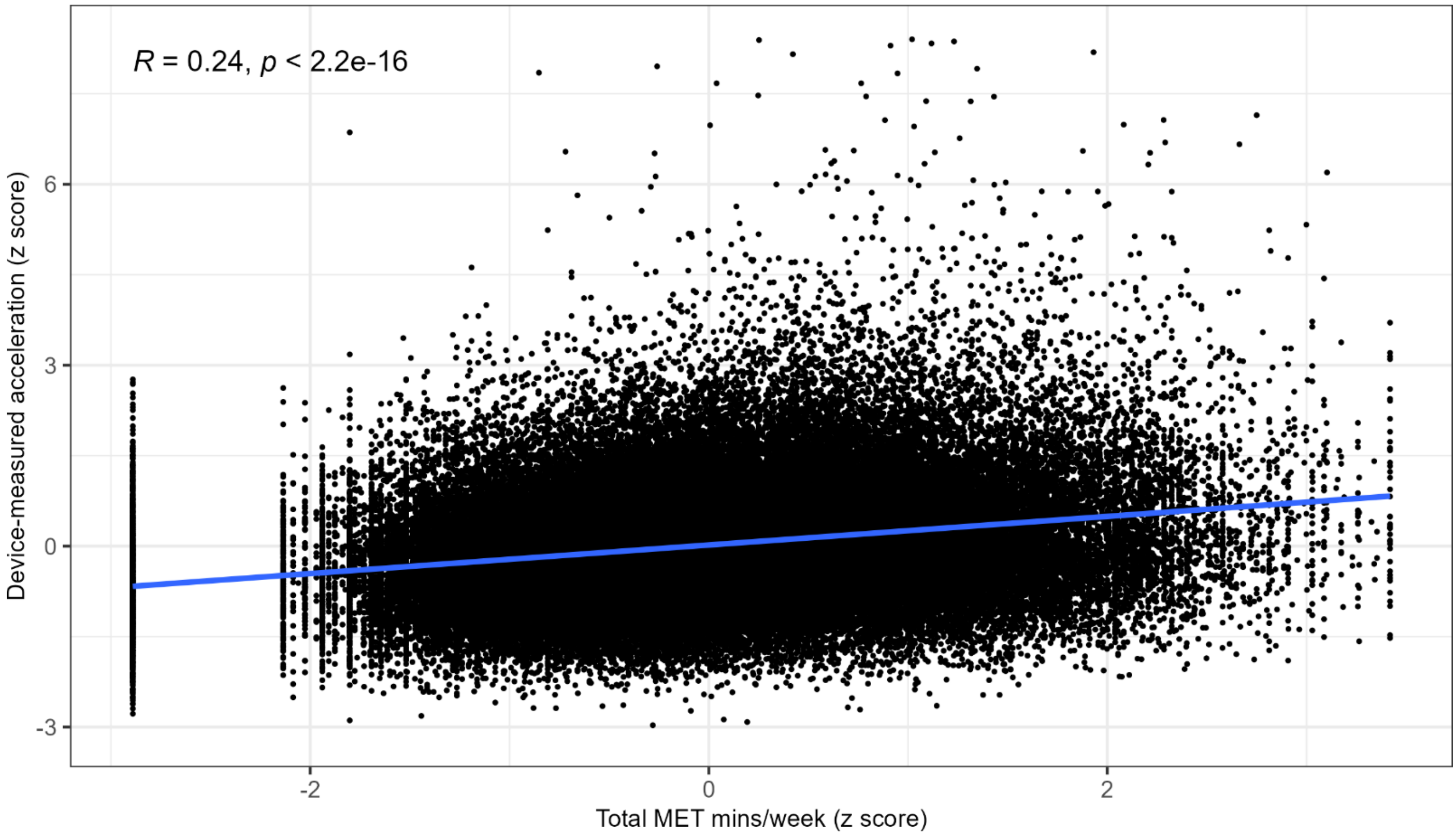

Supplementary Figure 1 - Association between questionnaire and accelerometer-derived overall activity measures. Scatter plot of questionnaire-derived total metabolic equivalent task (MET) minutes per week, and accelerometery-derived overall acceleration average (both normalised and centred). The blue line represents the fitted linear regression model.
R = Pearson correlation coefficient

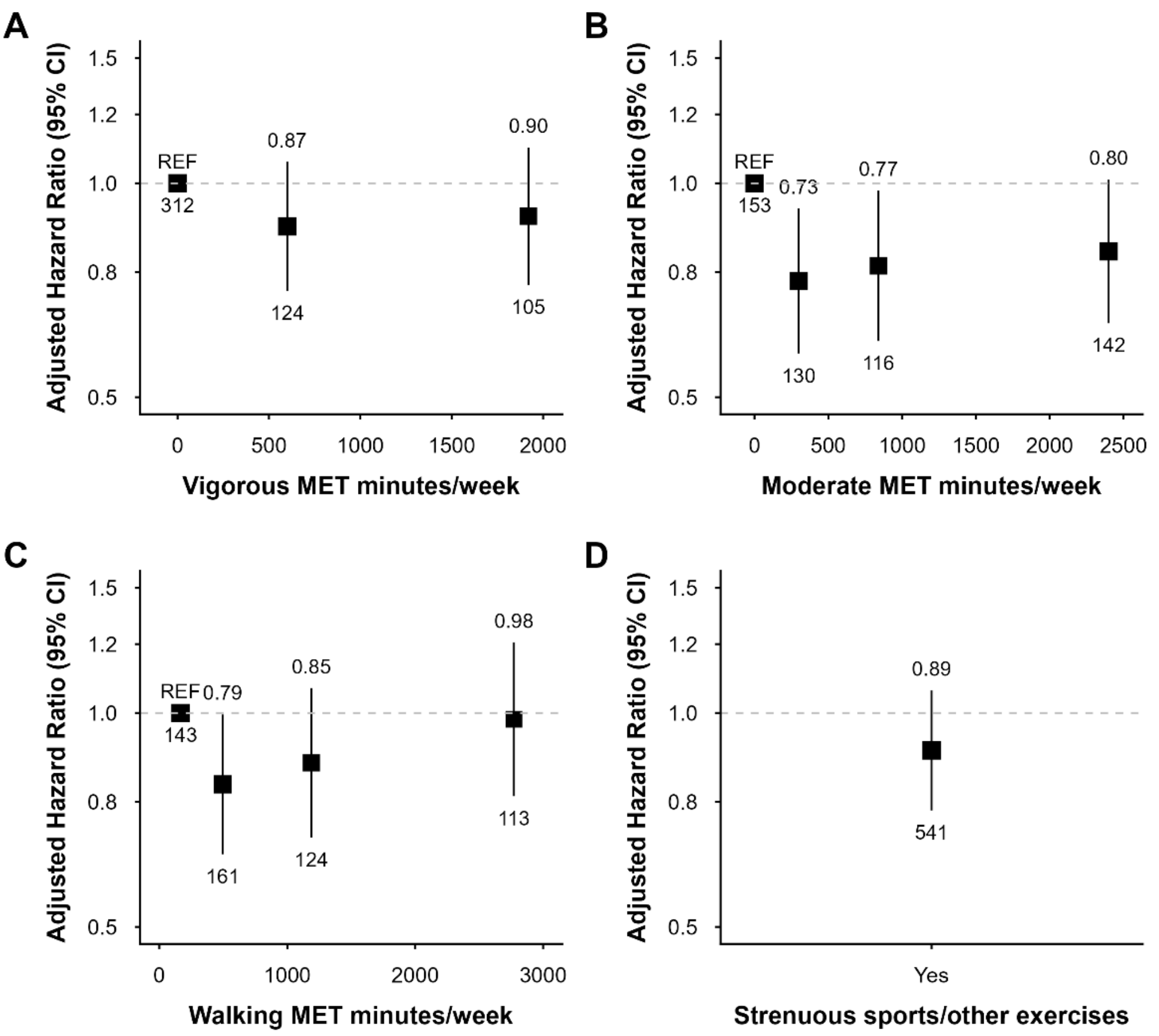

Supplementary Figure 2 - Association between questionnaire-derived activity measures and risk of incident ALS. Hazard ratios (HR) for ALS by quartiles of different intensity metabolic equivalent task (MET) minutes/week, calculated using Cox models with age as the timescale and adjusted for sex, employment status, age leaving education, Townsend Deprivation index, smoking status, alcohol consumption and body mass index (BMI). The number above each vertical line is the HR and the number below each vertical line is the number of events. "Strenuous sports and other exercises" is defined as any strenuous sports or other exercises at least 2-3 times per week for at least 15-30 mins, as classified by Julian et al[10]. The first and second quartiles of vigorous MET minutes/week are combined because the cut-off between the two is 0 minutes/week.

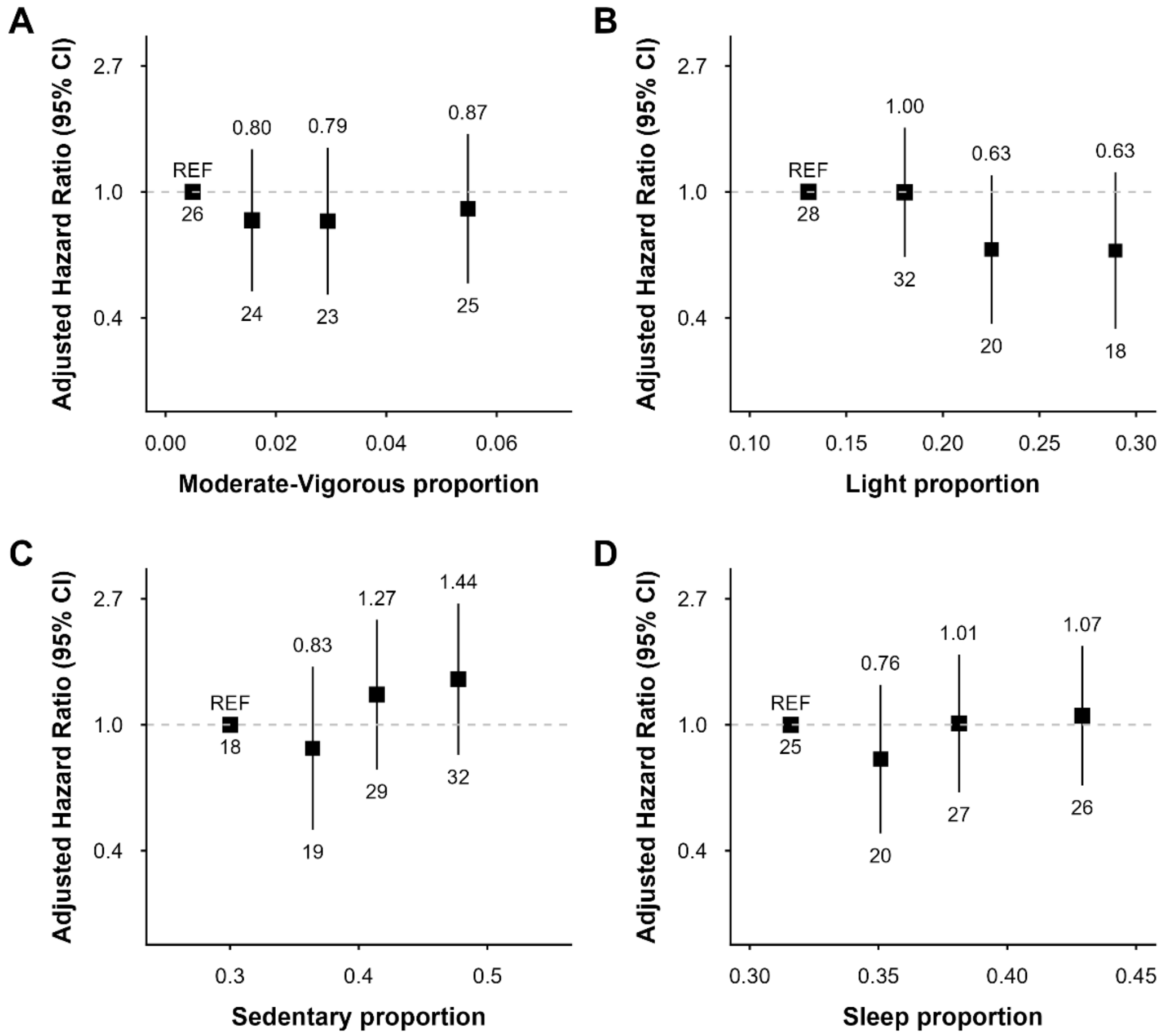

Supplementary Figure 3 - Association between accelerometer-derived activity measures and risk of incident ALS. Hazard ratios (HR) of ALS by quartiles of different accelerometer-derived activity measures, calculated using Cox models with age as the timescale and adjusted for sex, employment status, age leaving education, Townsend Deprivation index, smoking status, alcohol consumption and body mass index (BMI). The number above each vertical line is the HR and the number below each vertical line is the number of events. Moderate-vigorous, light, sedentary and sleep proportions were estimated using machine learning models by Willetts et al[11]

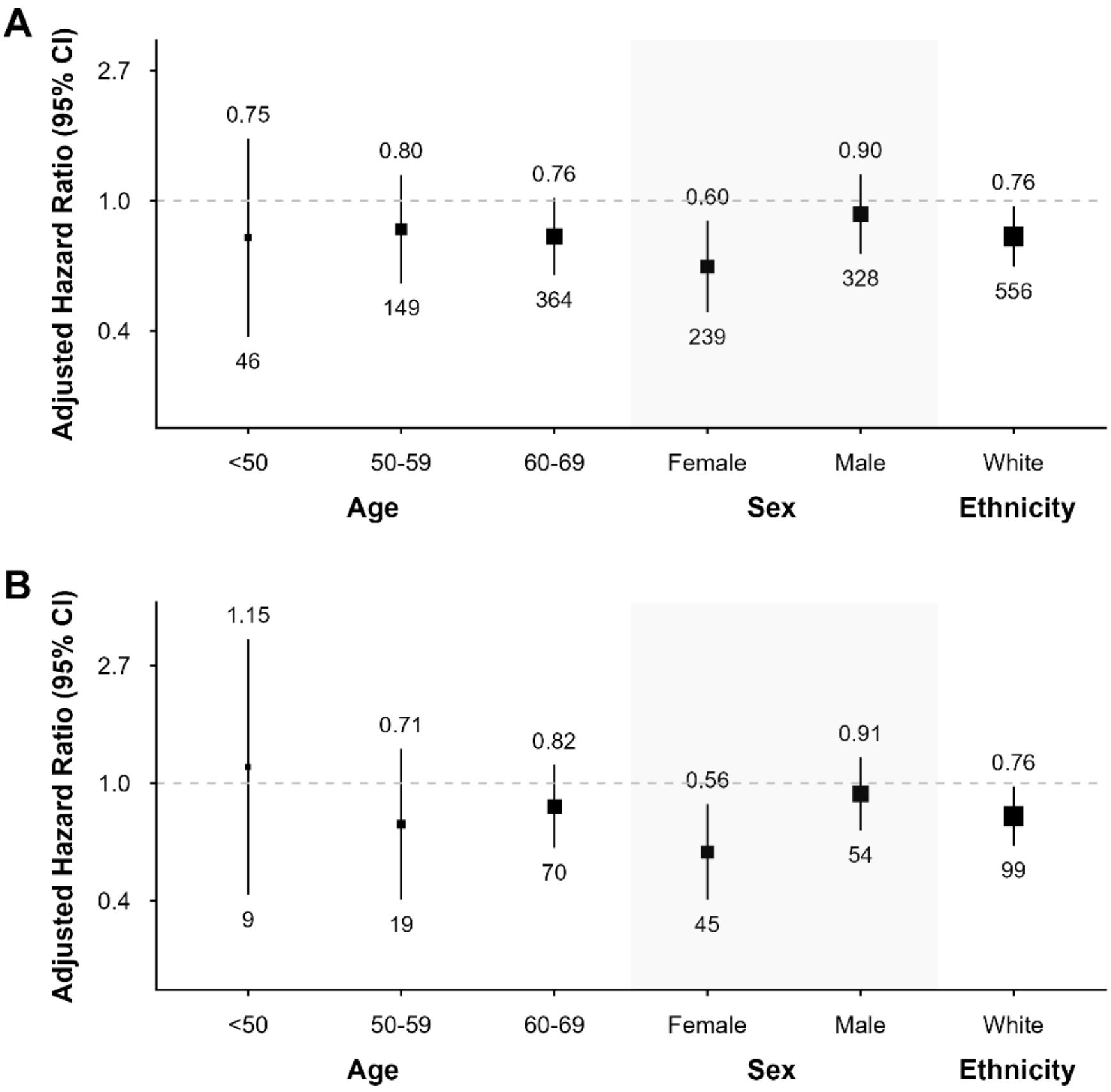

Supplementary Figure 4 - Analysis by age category, sex and ethnicity.
Hazard ratios (HR) of ALS for the self-reported IPAQ high category compared to low category (A) and per 1 SD change in device-measured overall acceleration (B), stratified by age category, sex and in individuals reporting white ethnicity. The 70+ age category is excluded due to insufficient numbers for analysis.

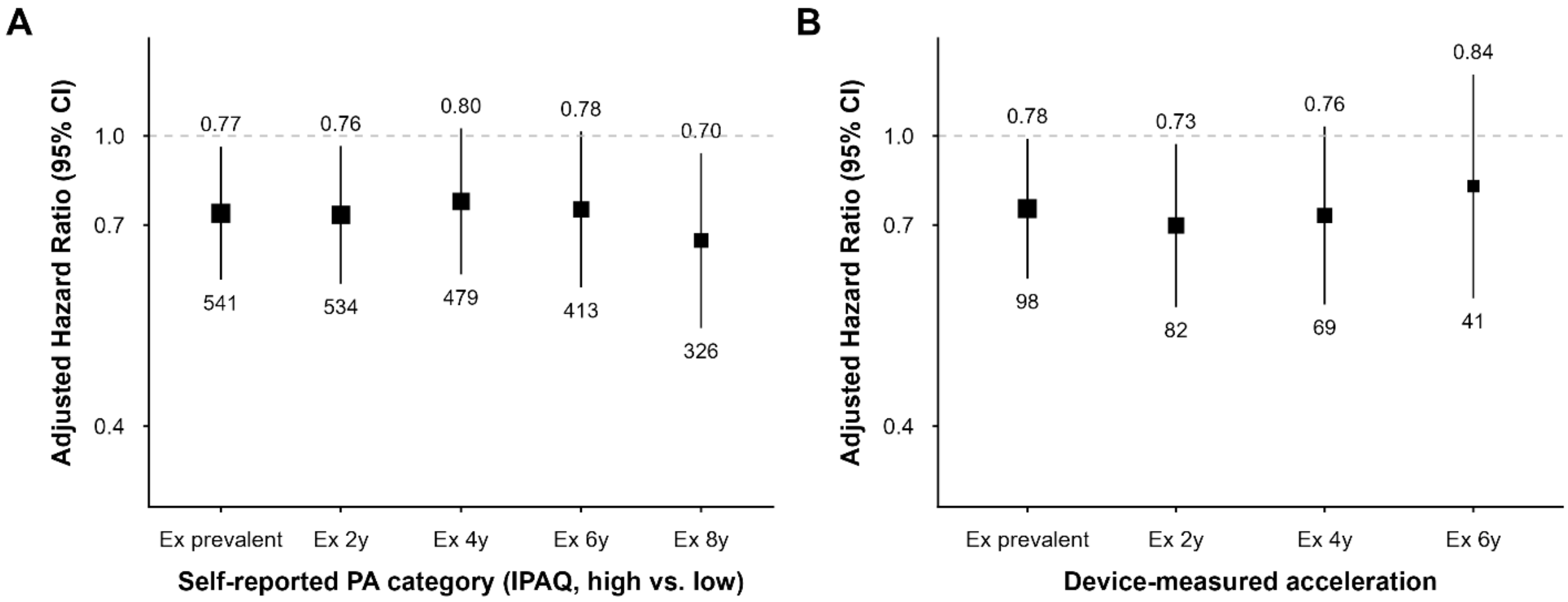

Supplementary Figure 5 - Effect of sequential exclusion of recently diagnosed ALS patients.
Hazard ratios (HR) of ALS for self-reported IPAQ high category compared to low category and per 1 SD change in device-measured overall acceleration with sequential exclusion of ALS cases recorded prior to, within 2 years, 4 years, 6 years and 8 years of activity measurement.

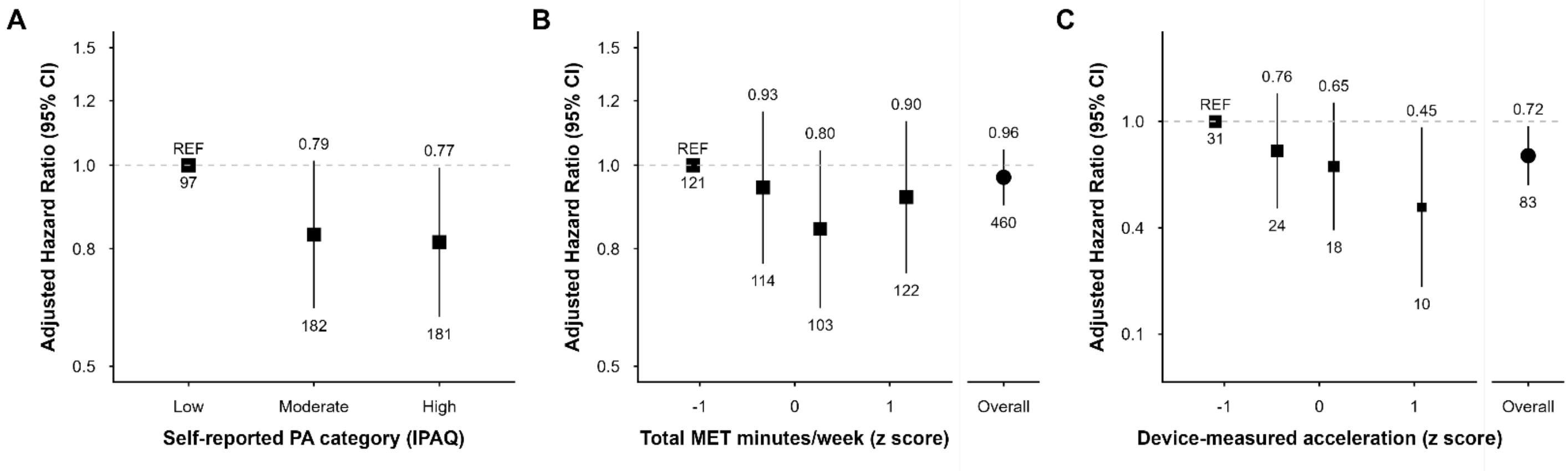

Supplementary Figure 6 - Analyses using only death certificate ALS diagnoses.
Hazard ratios for ALS by self-reported IPAQ physical activity category (A), quartiles of metabolic equivalent task (MET) minutes/week (B) quartiles of device-measured overall acceleration (C) and overall HR per 1 SD increase in each activity measure when treated as a continuous variable, using ALS diagnoses from death certificate data only.

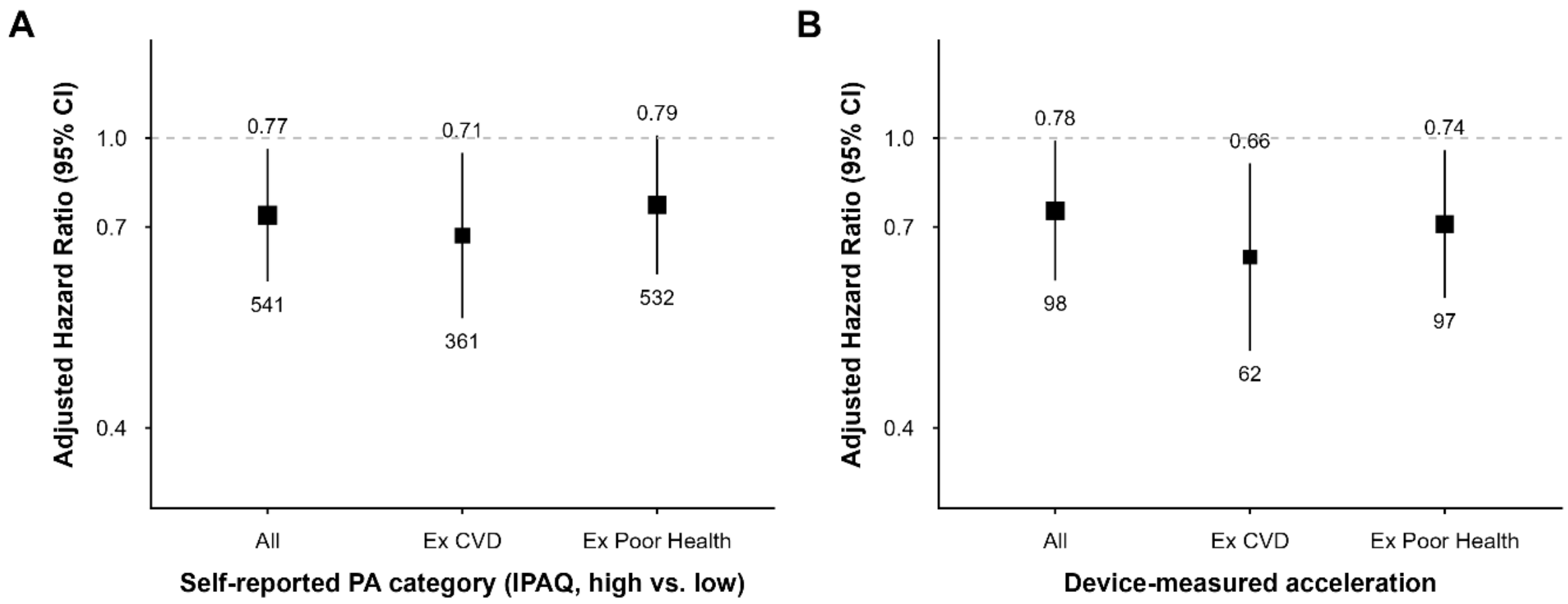

Supplementary Figure 7 - Effect of exclusion of participants with pre-existing cardiovascular disease or self-reported poor health.
Hazard ratios (HR) of ALS for self-reported IPAQ high category compared to low category (A) and per 1 SD change in device-measured overall acceleration (B) with exclusion of participants with pre-existing cardiovascular disease ("Ex CVD") or self-reported poor health ("Ex Poor Health").

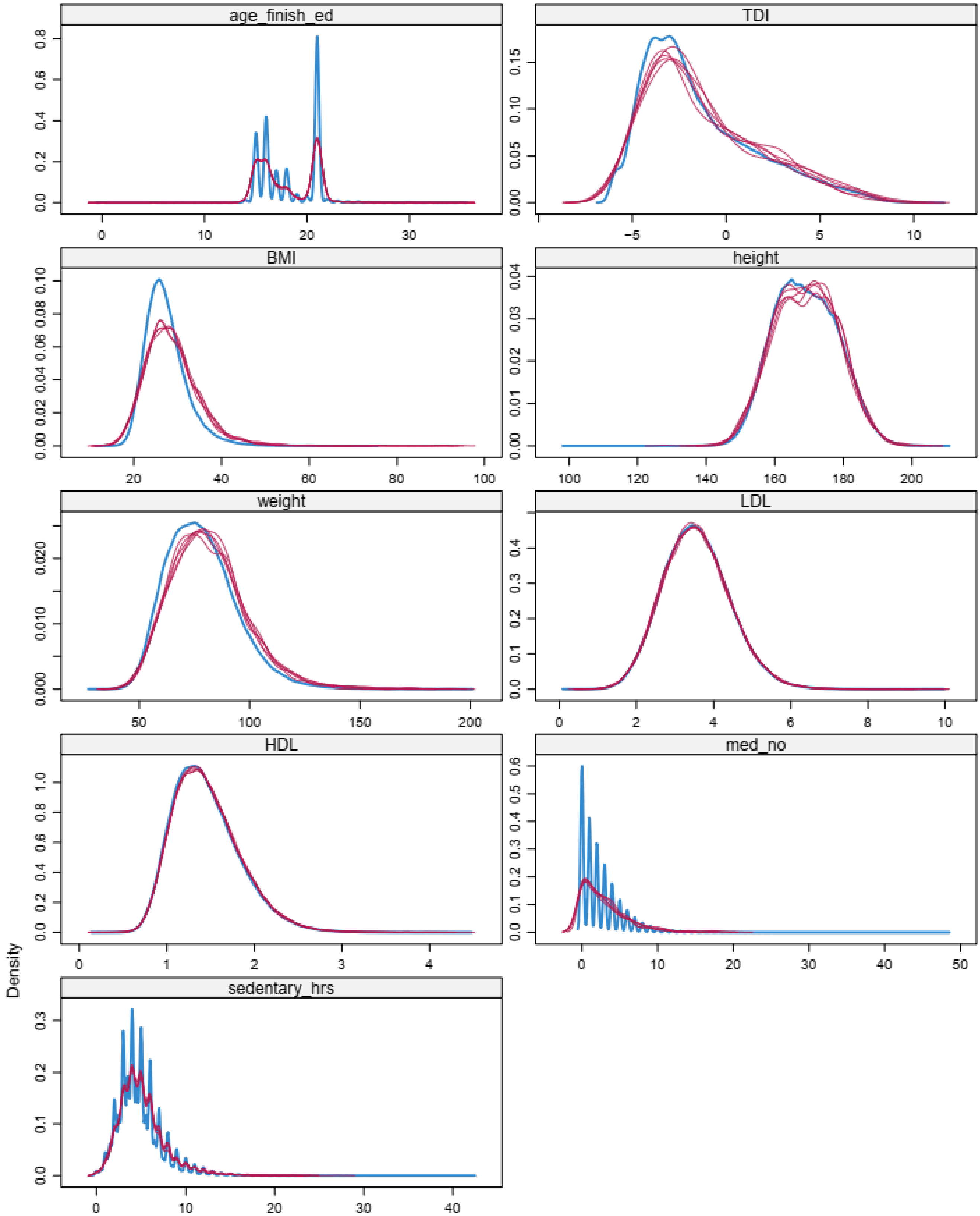

Supplementary Figure 8 – Density plot of continuous variables for the first 5 MICE imputations in individuals with complete exercise questionnaire data

Blue lines represent the densities of observed values, each pink line represents the densities of imputed values in one imputation. age_finish_ed – Age leaving full time education; TDI – Townsend deprivation index, med_no – number of medications

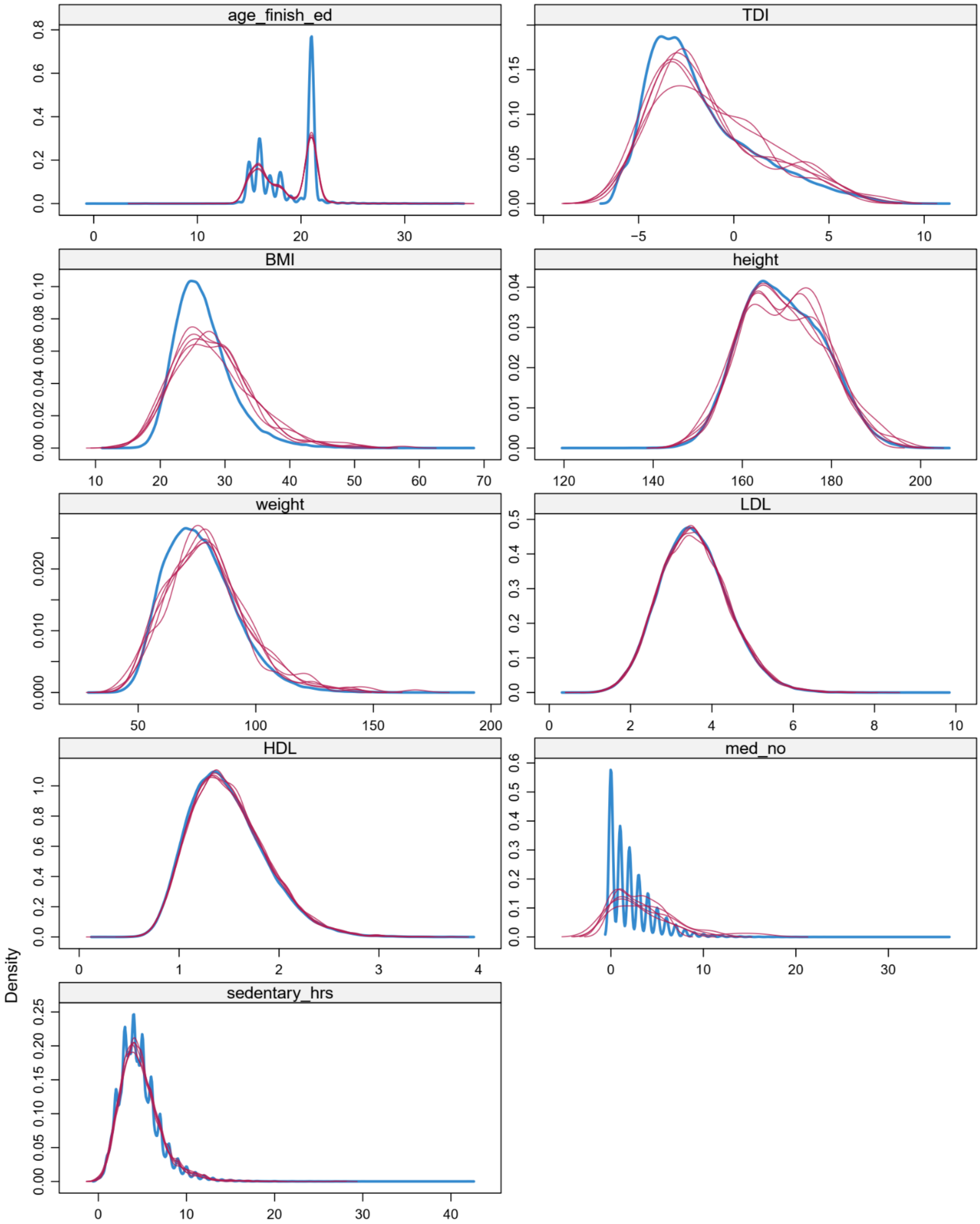

Supplementary Figure 9 – Density plot of continuous variables for the first 5 MICE imputations in individuals with complete accelerometery data

Blue lines represent the densities of observed values, each pink line represents the densities of imputed values in one imputation. age_finish_ed – Age leaving full time education; TDI – Townsend deprivation index, med_no – number of medications

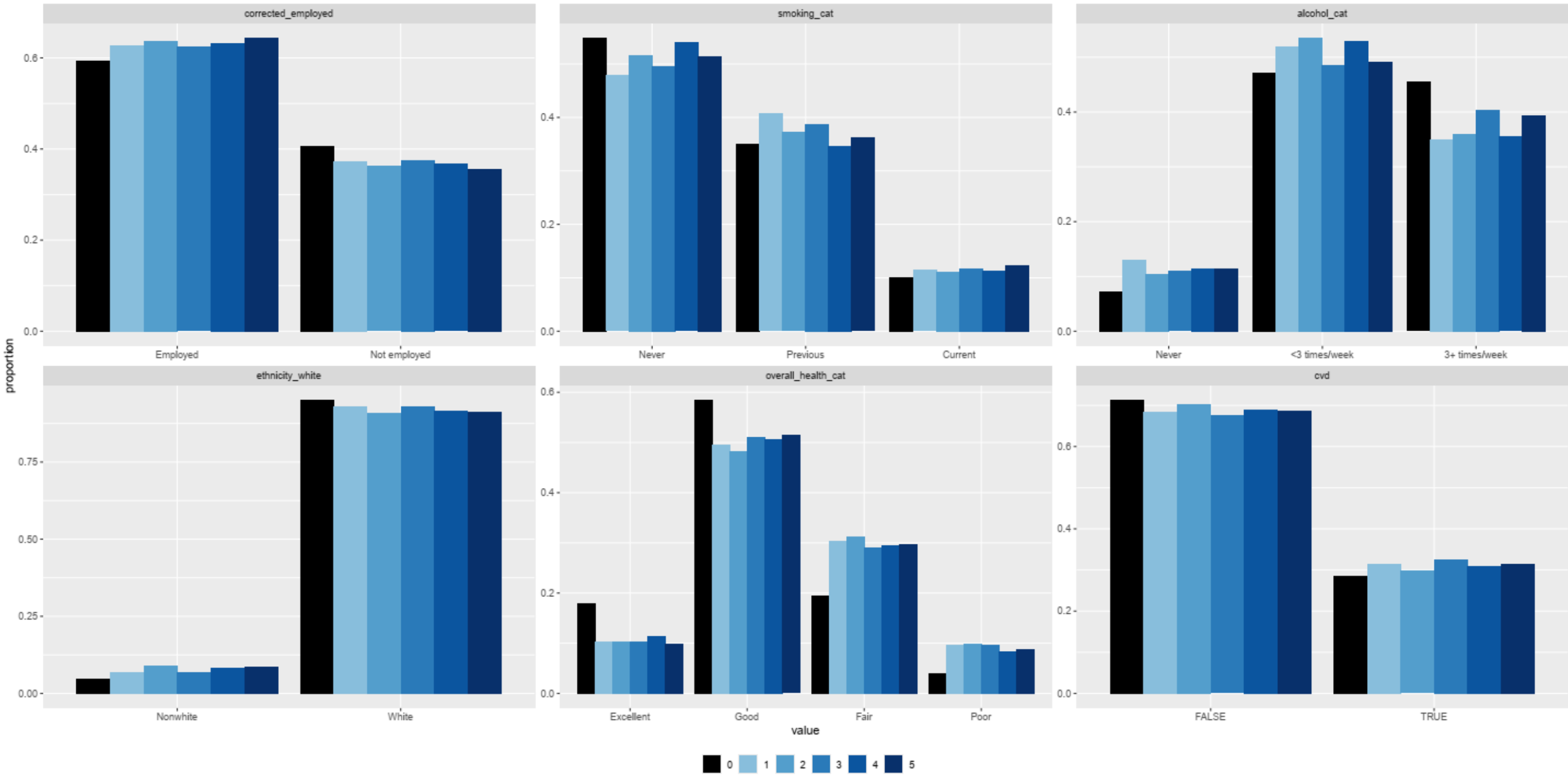

Supplementary Figure 10 – Proportion plot of categorical variables for the first 5 MICE imputations in individuals with complete exercise questionnaire data

The black bars (0) represent observed values and each blue bar (1-5) represents values from one imputation. corrected_employed – Employment status, smoking_cat – smoking status, alcohol_cat – alcohol consumption, ethnicity_white – ethnicity, overall_health_cat – overall health, cvd – cardiovascular disease

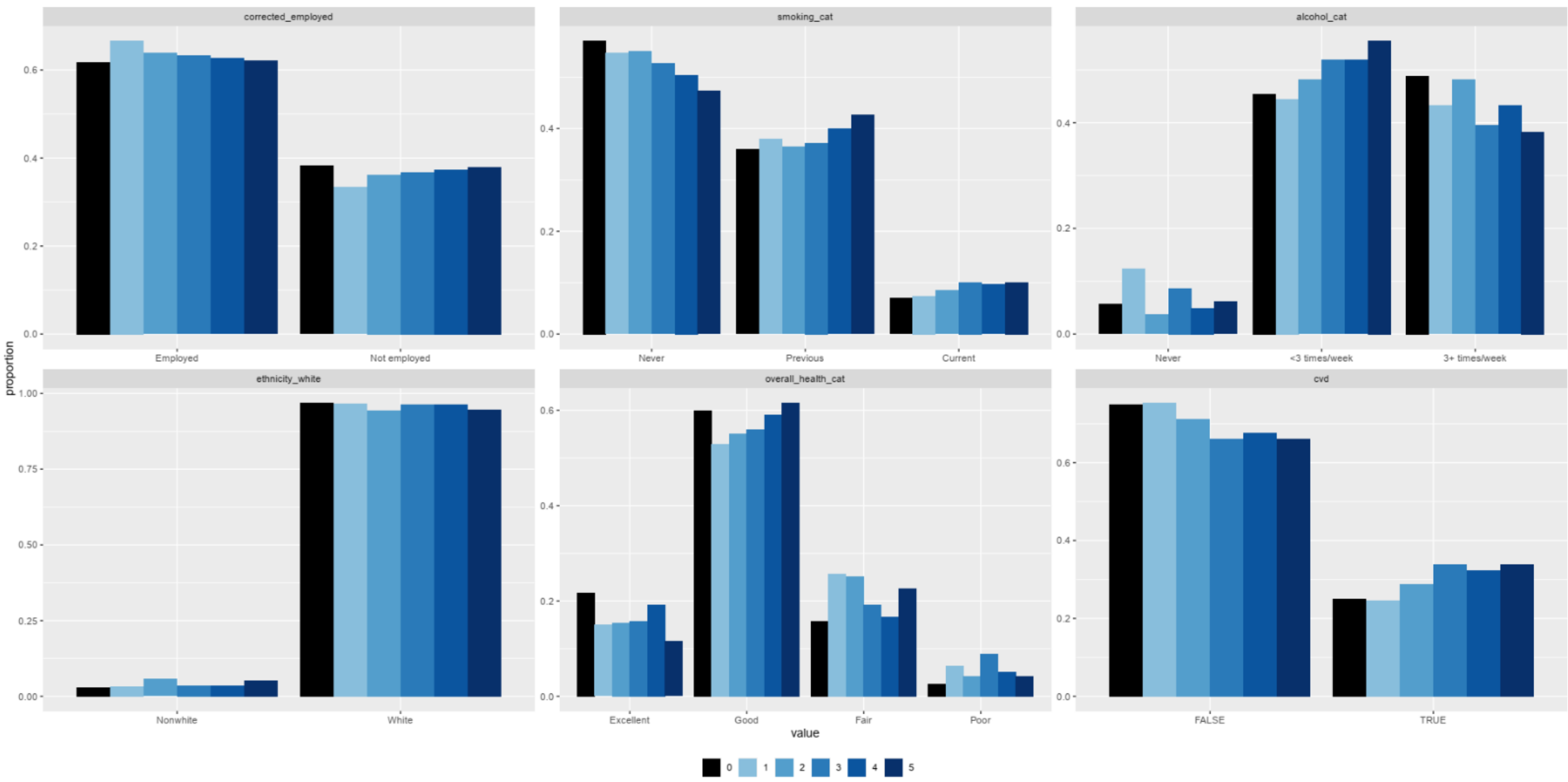

Supplementary Figure 11 – Proportion plot of categorical variables for the first 5 MICE imputations in individuals with complete accelerometery data

The black bars (0) represent observed values and each blue bar (1-5) represents values from one imputation. corrected_employed – Employment status, smoking_cat – smoking status, alcohol_cat – alcohol consumption, ethnicity_white – ethnicity, overall_health_cat – overall health, cvd – cardiovascular disease

References

1 Pate RR, Pratt M, Blair SN, *et al.* Physical activity and public health. A recommendation from the Centers for Disease Control and Prevention and the American College of Sports Medicine. *JAMA*. 1995;273:402–7. doi: 10.1001/jama.273.5.402

2 IPAQ. Guidelines for Data Processing and Analysis of the International Physical Activity Questionnaire (IPAQ). 2005.

3 Dolzhenko E, Vugt JJFA van, Shaw RJ, *et al.* Detection of long repeat expansions from PCR-free whole-genome sequence data. *Genome Res*. 2017;27:1895–903. doi: 10.1101/gr.225672.117

4 ExpansionHunter/variant_catalog/grch38 at master · Illumina/ExpansionHunter · GitHub. https://github.com/Illumina/ExpansionHunter/tree/master/variant_catalog/grch38 (accessed 22 June 2024)

5 Weidburd B, VanNoy G, Watts N. The Addition of Short Tandem Repeat Calls to gnomAD (v3.1.3). 2022. https://gnomad.broadinstitute.org/news/2022-01-the-addition-of-short-tandem-repeat-calls-to-gnomad/

6 Dolzhenko E, Weisburd B, Ibañez K, *et al.* REViewer: haplotype-resolved visualization of read alignments in and around tandem repeats. *Genome Medicine*. 2022;14:84. doi: 10.1186/s13073-022-01085-z

7 Pottier C, Ren Y, Perkerson RB, *et al.* Genome-wide analyses as part of the international FTLD-TDP whole-genome sequencing consortium reveals novel disease risk factors and increases support for immune dysfunction in FTLD. *Acta Neuropathol*. 2019;137:879–99. doi: 10.1007/s00401-019-01962-9

8 Machiela MJ, Chanock SJ. LDlink: a web-based application for exploring population-specific haplotype structure and linking correlated alleles of possible functional variants. *Bioinformatics*. 2015;31:3555–7. doi: 10.1093/bioinformatics/btv402

9 Rubin DB, Schenker N. Multiple imputation in health-care databases: an overview and some applications. *Stat Med*. 1991;10:585–98. doi: 10.1002/sim.4780100410

10 Julian TH, Glascow N, Barry ADF, *et al.* Physical exercise is a risk factor for amyotrophic lateral sclerosis: Convergent evidence from Mendelian randomisation, transcriptomics and risk genotypes. *EBioMedicine*. 2021;68:103397. doi: 10.1016/j.ebiom.2021.103397

11 Willetts M, Hollowell S, Aslett L, *et al.* Statistical machine learning of sleep and physical activity phenotypes from sensor data in 96,220 UK Biobank participants. *Sci Rep*. 2018;8:7961. doi: 10.1038/s41598-018-26174-1

STROBE Statement—checklist of items that should be included in reports of observational studies

	Item No.	Recommendation	Page No.	Relevant text from manuscript
**Title and abstract**	1	(*a*) Indicate the study's design with a commonly used term in the title or the abstract	2-3	
		(*b*) Provide in the abstract an informative and balanced summary of what was done and what was found	2-3	
**Introduction**				
Background/rationale	2	Explain the scientific background and rationale for the investigation being reported	4-5	
Objectives	3	State specific objectives, including any prespecified hypotheses	5	
**Methods**				
Study design	4	Present key elements of study design early in the paper	5-7	
Setting	5	Describe the setting, locations, and relevant dates, including periods of recruitment, exposure, follow-up, and data collection	5-7 and supplementary methods	
Participants	6	(*a*) *Cohort study*—Give the eligibility criteria, and the sources and methods of selection of participants. Describe methods of follow-up<br>*Case-control study*—Give the eligibility criteria, and the sources and methods of case ascertainment and control selection. Give the rationale for the choice of cases and controls<br>*Cross-sectional study*—Give the eligibility criteria, and the sources and methods of selection of participants	5-6	
		(*b*) *Cohort study*—For matched studies, give matching criteria and number of exposed and unexposed<br>*Case-control study*—For matched studies, give matching criteria and the number of controls per case	NA	
Variables	7	Clearly define all outcomes, exposures, predictors, potential confounders, and effect modifiers. Give diagnostic criteria, if applicable	5-7 and supplementary methods	

Data sources/ measurement	8*	For each variable of interest, give sources of data and details of methods of assessment (measurement). Describe comparability of assessment methods if there is more than one group	5-7 Supp Table1
Bias	9	Describe any efforts to address potential sources of bias	7-9
Study size	10	Explain how the study size was arrived at	NA
Quantitative variables	11	Explain how quantitative variables were handled in the analyses. If applicable, describe which groupings were chosen and why	7-9 and supplementary methods
Statistical methods	12	(*a*) Describe all statistical methods, including those used to control for confounding	7-9
		(*b*) Describe any methods used to examine subgroups and interactions	7-9
		(*c*) Explain how missing data were addressed	7-9 and supplementary methods
		(*d*) *Cohort study*—If applicable, explain how loss to follow-up was addressed<br>*Case-control study*—If applicable, explain how matching of cases and controls was addressed<br>*Cross-sectional study*—If applicable, describe analytical methods taking account of sampling strategy	7-9
		(*e*) Describe any sensitivity analyses	7-9
**Results**			
Participants	13*	(a) Report numbers of individuals at each stage of study—eg numbers potentially eligible, examined for eligibility, confirmed eligible, included in the study, completing follow-up, and analysed	9-11
		(b) Give reasons for non-participation at each stage	9-11
		(c) Consider use of a flow diagram	NA
Descriptive data	14*	(a) Give characteristics of study participants (eg demographic, clinical, social) and information on exposures and potential confounders	Table 1
		(b) Indicate number of participants with missing data for each variable of interest	Supp Table 3
		(c) *Cohort study*—Summarise follow-up time (eg, average and total amount)	9

Outcome data	15*	*Cohort study*—Report numbers of outcome events or summary measures over time	9-12 and in figures
		Case-control study—Report numbers in each exposure category, or summary measures of exposure	
		Cross-sectional study—Report numbers of outcome events or summary measures	
Main results	16	(*a*) Give unadjusted estimates and, if applicable, confounder-adjusted estimates and their precision (eg, 95% confidence interval). Make clear which confounders were adjusted for and why they were included	9-12 and in figures
		(*b*) Report category boundaries when continuous variables were categorized	9-12 and in figures
		(*c*) If relevant, consider translating estimates of relative risk into absolute risk for a meaningful time period	NA
Other analyses	17	Report other analyses done—eg analyses of subgroups and interactions, and sensitivity analyses	11-12
**Discussion**			
Key results	18	Summarise key results with reference to study objectives	12-13
Limitations	19	Discuss limitations of the study, taking into account sources of potential bias or imprecision. Discuss both direction and magnitude of any potential bias	14-15
Interpretation	20	Give a cautious overall interpretation of results considering objectives, limitations, multiplicity of analyses, results from similar studies, and other relevant evidence	13-15
Generalisability	21	Discuss the generalisability (external validity) of the study results	13-15
**Other information**			
Funding	22	Give the source of funding and the role of the funders for the present study and, if applicable, for the original study on which the present article is based	15-16

*Give information separately for cases and controls in case-control studies and, if applicable, for exposed and unexposed groups in cohort and cross-sectional studies.
